# Complete Genome Sequence and Comparative Genome Analysis of *Variovorax* sp. Strains PAMC28711, PAMC26660, and PAMC28562 and Trehalose Metabolic Pathways in Antarctica Isolates

**DOI:** 10.1155/2022/5067074

**Published:** 2022-11-09

**Authors:** Prasansah Shrestha, Jayram Karmacharya, So-Ra Han, Jun Hyuck Lee, Hyun Park, Tae-Jin Oh

**Affiliations:** ^1^Department of Life Science and Biochemical Engineering, Graduate School, Sun Moon University, Asan 31460, Republic of Korea; ^2^Unit of Research for Practical Application, Korea Polar Research Institute, Incheon 21990, Republic of Korea; ^3^Department of Polar Sciences, University of Science and Technology, Incheon 21990, Republic of Korea; ^4^Division of Biotechnology, College of Life Sciences and Biotechnology, Korea University, Seoul 02841, Republic of Korea; ^5^Genome-Based Bio IT Convergence Institute, Asan 31460, Republic of Korea; ^6^Department of Pharmaceutical Engineering and Biotechnology, Sun Moon University, Asan 31460, Republic of Korea

## Abstract

The complete genomes of *Variovorax* strains were analyzed and compared along with the genomes of *Variovorax* strains PAMC28711, PAMC28562, and PAMC26660, Antarctic isolates. The genomic information was collected from the NCBI database and the CAZyme database, and Prokka annotation was used to find the genes that encode for the trehalose metabolic pathway. Likewise, CAZyme annotation (dbCAN2 Meta server) was performed to predict the CAZyme family responsible for trehalose biosynthesis and degradation enzymes. Trehalose has been found to respond to osmotic stress and extreme temperatures. As a result, the study of the trehalose metabolic pathway was carried out in harsh environments such as the Antarctic, where bacteria *Variovorax* sp. strains PAMC28711, PAMC28562, and PAMC26660 can survive in extreme environments, such as cold temperatures. The trehalose metabolic pathway was analyzed via bioinformatics tools, such as the dbCAN2 Meta server, Prokka annotation, Multiple Sequence Alignment, ANI calculator, and PATRIC database, which helped to predict trehalose biosynthesis and degradation genes' involvement in the complete genome of *Variovorax* strains. Likewise, MEGA *X* was used for evolutionary and conserved genes. The complete genomes of *Variovorax* strains PAMC28711, PAMC26660, and PAMC28562 are circular chromosomes of length (4,320,000, 7,390,000, and 4,690,000) bp, respectively, with GC content of (66.00, 66.00, and 63.70)%, respectively. The GC content of these three *Variovorax* strains is lower than that of the other *Variovorax* strains with complete genomes. Strains PAMC28711 and PAMC28562 exhibit three complete trehalose biosynthetic pathways (OtsA/OtsB, TS, and TreY/TreZ), but strain PAMC26660 only possesses one (OtsA/OtsB). Despite the fact that all three strains contain trehalose, only strain PAMC28711 has two trehalases according to CAZyme families (GH37 and GH15). Moreover, among the three Antarctica isolates, only strain PAMC28711 exhibits auxiliary activities (AAs), a CAZyme family. To date, although the *Variovorax* strains are studied for different purposes, the trehalose metabolic pathways in *Variovorax* strains have not been reported. Further, this study provides additional information regarding trehalose biosynthesis genes and degradation genes (trehalases) as one of the factors facilitating bacterial survival under extreme environments, and this enzyme has shown potential application in biotechnology fields.

## 1. Introduction

*Variovorax* is a Gram-negative and motile bacterium belonging to the family Comamonadaceae [1] that is found in a straight to slightly curved or rod-shaped form. *Variovorax* colonies are yellow due to the presence of carotenoid pigments, and their colonies are slimy and shiny on nutrient agar. Many strains belonging to the family Comamonadaceae thrive in polluted environments and degrade complex organic compounds [2], whereas *Variovorax* generally inhabits soil and water [3]. *Variovorax* sp. PAMC28711 [4], *Variovorax* sp. PAMC26660, and *Variovorax* sp. PAMC28562 were isolated from Antarctica, and they are complete metagenomic assembled genomes.

According to the Pearce group, due to the size of Antarctica, there are many other specific extremes, such as areas with volcanic activity, hypersaline lakes, subglacial lakes, and even inside the ice itself, for which specialized extremophiles may be adapted [5]. Therefore, numerous microorganisms have specifically adapted to a wide range of extreme environments to survive in novel biodiversity, much of which has yet to be elucidated [3]. Another key feature of the Antarctic ecosystem is the extreme variation in the physical conditions, ranging from freshwater lakes (some of the most oligotrophic environments on Earth) to hypersaline lakes [6]. Microorganisms found under extreme environmental conditions like Antarctica are ideal candidates for the study of eco-physiological and biochemical adaptations of such life forms [5]. Antarctica is one of the most physically and chemically challenging terrestrial environments for habitation [7]. Habitats with permanently low temperatures dominate the temperate biosphere and have been successfully colonized by a wide variety of organisms that are collectively termed psychrophiles or cold-adapted organisms [8]. Lichens are characterized by a mutualistic symbiosis between fungi and photosynthetic algae or cyanobacteria, but they also have other associated bacterial communities [9]. Bacteria associated with lichens were initially reported in the first half of the 20^th^ century [10]. The lichen-associated microorganism was reported to carry genes involved in the degradation of polymers [11].

CAZymes belong to a large class of enzymes that are involved in the synthesis and degradation of complex carbohydrates. Based on their amino acid sequences, they are classified into families with conserved catalytic mechanism, structure, and active site residues but differ in substrate specificity [12]. They are responsible for carbohydrate synthesis through glycosyltransferases (GTs), degradation of complex carbohydrates via glycoside hydrolases (GHs), polysaccharide lyases (PLs), carbohydrate esterases (CEs), and enzymes for auxiliary activities (AAs) and recognition (carbohydrate-binding module, CBM) [13]. The GHs are the largest family of CAZymes that hydrolyze the glycosidic bond between two or more carbohydrates, or between carbohydrate and noncarbohydrate moieties, via the overall inversion of anomeric carbon [14].

Although the trehalose metabolism has been studied in various microorganisms, it has yet to be elucidated in the genomes of *Variovorax*. The sp. belonging to the phylum proteobacteria is able to degrade complex carbohydrates after Bacteroidetes and Firmicutes [15]. Even so, the disaccharide (such as Trehalose) utilization ability of the genus *Variovorax* has not previously been highlighted. Therefore, this study compares trehalose metabolic pathway in cold-adapted *Variovorax* strains PAMC28711, PAMC26660, and PAMC28562 acquired from the Antarctic region with other complete genomes of *Variovorax* strains deposited in the NCBI until October 2021. In addition, the study also covers the genes that encode for different CAZy families involved in the trehalose metabolic pathway in the complete genomes of *Variovorax* along with our three strains isolated from the Antarctic region. Bioinformatics tools like dbCAN, RAST, PATRIC database, KEGG pathway database, and Prokka annotation standalone program can assist in the prediction of trehalose synthesis and degradation genes' involvement of the microorganisms for preliminary screening approach without any experimental work.

## 2. Materials and Methods

### 2.1. Isolation, Genomic DNA Extraction, Genome Sequencing, and Assembly of the Complete Genomes of *Variovorax* sp. PAMC28711, PAMC28562, and PAMC26660

*Variovorax* sp. strains PAMC 28711, PAMC28562, and PAMC26660 (deposited as PAMC28711, PAMC28562, and PAMC26660 in the Polar and Alpine Microbial Collection, Korea Polar Research Institute, Incheon, Korea) were isolated from an Antarctic specimen collected on Barton Peninsula, King George Island, Antarctica (62°13′S, 58°47′W). *Variovorax* sp. strain PAMC28711 genome annotation was reported from our group in their previous result [4]. A pure R2A agar was used to isolate the bacterial sample for DNA analysis at 15°C. Using a QIAamp DNAMini Kit (Qiagen, Valencia, CA), genomic DNA was extracted from *Variovorax* sp. PAMC28562 and PAMC26660, and the quantity and purity were evaluated by the Agilent 2100 Bioanalyzer (Agilent Technologies, Santa Clara, CA). To assess the quality of the isolated DNA, agarose gel electrophoresis was used. DNA was kept at −20°C until required. PacBio sequel single-molecule real-time (SMRT) sequencing technology was used to sequence the genome (Pacific Biosciences, Menlo Park, CA). SMRT cells were used to sequence SMRbell library inserts (20 Kb). The strains PAMC26660 and PAMC28562 were used to extract raw sequence data of (7,388,698 and 4,693,528) bp, respectively. These were assembled *de novo* using the hierarchical genome-assembly process (HGAP v.4) protocol [16] and HGAP4 assembly using Pacific Biosciences' SMRT analysis software (version 2.3) (https://github.com/PacificBiosciences/SMRT-Analysis). The complete genome sequences for PAMC26660 and PAMC28562 were deposited in the GenBank database under the accession numbers NZ_CP060295 and NZ_CP060296, respectively.

### 2.2. Genome Annotation of *Variovorax* sp

The genomes of strains PAMC28711, PAMC28562, and PAMC26660 were annotated using the rapid annotation subsystem technology (RAST) server [17] and Prokka annotation [18]. For comparative studies, data on the *Variovorax* complete genomes were obtained from the National Center for Biotechnology Information (NCBI) database and PATRIC database [19]. The enzymes involved in the trehalose metabolism pathways were determined using the Kyoto Encyclopedia of Genes and Genomes (KEGG) database and a 0.01 cutoff value [20]. CAZyme gene analysis was performed using the dbCAN program [21] and a hidden model (HMM) profile retrieved from the dbCAN2 HMMdb database (version 7.0). Simultaneously, we obtained information regarding the existence of CAZyme genes from Signal P (version 4.0) [22]. The coverage criteria were >0.35, and the e-value cutoff was 1*e* − 15. To maximize prediction accuracy, we applied DIAMOND [23] (*e*-value 1*e*102) and Hotpep [24] (frequency >2.6, hits >6).

### 2.3. Complete Nucleotide Sequence and Strain Accession Numbers

The complete nucleotide sequences of *Variovorax* sp. strains PAMC28562 and PAMC26660 were deposited in the GenBank database under the accession numbers CP060296 and CP060295, respectively.

### 2.4. Phylogenomic Classification and Average Nucleotide Identity (ANI) of *Variovorax* sp. PAMC28711, PAMC26660, and PAMC28562

The genomes of *Variovorax* sp. strains PAMC28711, PAMC28562, and PAMC26660 were uploaded to the Type (Strain) Genome Server (TYGS) [25] for whole-genome-based taxonomic analysis [26]. The genomes of the closest type strains were determined in two ways: first, the genomes of the PAMC28711, PAMC26660, and PAMC28562 strains were compared to all the type strain genomes available in the TYGS database using the MASH algorithm, a fast approximation of intergenomic relatedness [27], and the type strains with the smallest MASH distances were chosen per genome of the PAMC28711, PAMC26660, and PAMC28562. Second, the 16S rDNA gene sequences were used to identify an additional group of closely related type strains. RNAmmer [28] was used to extract these sequences from the genomes of the PAMC28711, PAMC26660, and PAMC28562 strains, and each sequence was then BLASTed [29] against the 16S rDNA gene sequences of each of the 11,252 type strains now accessible in the TYGS database. The pairwise comparison of the user strain with the type strains was performed using GBDP, and accurate intergenomic distances were inferred under the “trimming” algorithm and distance formula d5. Digital DDH values and confidence intervals were calculated following the recommended settings of GGDC 2.1 [26] The intergenomic distances were used to create a balanced minimum evolution tree using FASTME 2.1.4 with 100 pseudo bootstrap replicates for branch support [30]. ANI analysis was performed using three different methods, like Orthologous Average Nucleotide Identity Software Tool (OAT) [31], JSp.WS [32], and FastANI [33].

### 2.5. Comparative Genomics Analysis

All strains of the complete genomes of *Variovorax* deposited in the NCBI database (https://www.ncbi.nlm.nih.gov/) until October 2021 were analyzed. First, we determined the relationship of PAMC28711, PAMC26660, and PAMC28562 with other strains from the same genus using complete genome sequences and checked their similarity by comparing the phylogenomic analysis. And then we have done the comparison of CAZymes from the registered sp. were referenced using bioinformatics tools, such as CAZyme annotation (dbCAN2 meta server; https://bcb.unl.edu/dbCAN2/), as well as using CAZy (https://www.cazy.org/). The Prokka annotation standalone program (https://vicbioinformatics.com) and the NCBI database were also used to find the genes that encode trehalose biosynthesis and degradation. The dbCAN2 meta server program annotates the genomes using DIAMOND, HMMER, and Hotpep via CAZy, dbCAN, and PPR databases [21]. The dbCAN2 meta server allows the submission of nucleotide sequences for prokaryotic and eukaryotic genomes, although protein sequences are preferred. This server uses three tools that comprise DIAMOND (for fast blast hits in the CAZy database), HMMER (for annotated CAZyme domain boundaries according to the dbCAN CAZyme domain HMM database), and Hotpep (for conserved short motifs in the PPR library). The Kyoto Encyclopedia of Genes and Genomics (KEGG) pathway database and the Prokka annotation standalone program were used to analyze the trehalose metabolic pathways of strains [18, 20, 34]. Likewise, the PATRIC database (https://patricbrc.org/) [19] was also used for genomic information.

### 2.6. Various Polysaccharides Screening of Strain PAMC28711 by AZCL Activity

We confirmed the activity through azurine cross-linked (AZCL) analysis, which is based on the visible solubilization of small particles of the AZCL polysaccharide substrate for CAZyme function activity. Seven AZCL substrates (AZCL-amylose, AZCL-barley *β*-glucan, AZCL-arabinoxylan, AZCL-HE-cellulose, AZCL-xylan (beech wood), AZCL-xylan (birch wood), and AZCL-xyloglucan), were used to determine the enzyme activity of the polysaccharide degradation in strain PAMC28711. This assay showed the formation of blue haloes around the well in agar media, indicating polysaccharide degrading activity [35]. PAMC28711 was incubated in four different media like Bennett's media (B's), Marine agar (MA), Malt Yeast (MY) media, and Reasoner's 2A agar (R2A) to detect active CAZyme-producing strains specifically and rapidly. The active culture plate consisted of 2% agarose, 25 mM sodium phosphate buffer (pH 5.5), and xanthan gum solidified in the plate. A total of 20 *μ*L of the original strain was dispensed on AZCL plates. The plates were incubated at different temperatures (4°C, 15°C, 25°C, and 37°C) for 7 to 10 days, and a blue halo was recorded to confirm activity. The AZCL activity was performed using a commercial kit from Megazyme© (Bray, Ireland; https://www.megazyme.com/) at different temperatures of (4, 15, 25, and 37)°C and expressed as the area (cm^2^) with a blue halo around the sample well in the AZCL assays [36, 37].

## 3. Results

### 3.1. Genomic Information of Genus *Variovorax* sp. PAMC28711, PAMC26660, and PAMC28562

The complete genome of *Variovorax* sp. strains PAMC28711, PAMC26660, and PAMC28562 is composed of circular chromosomes of (4,320,000, 7,390,000, and 4,690,000) bp, respectively, with GC contents of (66.00, 66.00, and 63.70)%, respectively. These three *Variovorax* strains have the lowest GC content, compared with those of other complete genomes of *Variovorax* strains (Table 1 and Supplementary Table S1). 4232, 6919, and 4402 genes were predicted on the chromosome in the strains PAMC28711, PAMC26660, and PAMC28562, with 4071, 6801, and 4298 protein-encoding genes functionally assigned and the rest predicted as hypothetical proteins. In the genomes of strains PAMC28711, PAMC26660, and PAMC28562, we found 106, 57, and 48 pseudogenes and 46, 52, and 47 tRNA genes, respectively (Supplementary Table S1).

### 3.2. Phylogenomic Classification and ANI Analysis of *Variovorax* Strains

**Figure 1:**
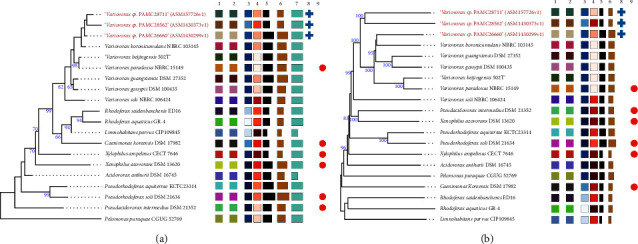
TYGS results for the *Variovorax* dataset. Tree inferred with FASTME 2.1.6 from GBDP distances calculated from (a) 16S rDNA gene sequences and (b) whole genome sequences. Branch lengths are scaled in terms of GBDP distance formula d5; the numbers above the branches are GBDP pseudo-bootstrap support values from 100 replicons. Leaf labels are annotated by affiliation to sp. cluster (1), subsp. cluster (2), GC% content (3), delta statistics (4), genome size (bp) (5), protein count (6), SSU length (bp) (7), user provided GenBank accession IDs are shown in parentheses (8), and type sp. (9).

The relationship between strains PAMC28711, PAMC26660, and PAMC28562 and their associated type strains was shown via a phylogenetic tree derived from the intergenomic distance measured using GBDP on the TYGS database (Figure 1). Based on the 16S rDNA comparison, strains PAMC28711 and PAMC28562 were found to be in the same node, while strain PAMC26660 was found in a different node. These three strains were found to be closest to the type strains *V. boronicumulans* NBRC 103145^T^, *V. beijingensis* 502^T^, and *V. paradoxus* NBRC 15149^T^ (Figure 1(a)), sharing the same clade. Likewise, the whole-genome-based phylogeny revealed a cluster of the same sp. as the closest relatives of PAMC28711, PAMC26660, and PAMC28562 (Figure 1(b)).

Finally, taxogenomic analyses of closely related sp. were performed, with the overall genome-related indexes calculated. The TYGS web server (https://tygs.dsmz.de/) and JSp.WS server (https://jsp.ribohost.com/jsp.ws) were used to perform digital DNA-DNA hybridization (dDDH) and average nucleotide identity (ANI) tests. The dDDH results showed 24.5%, 24.3%, and 24.5% for PAMC28711, 31.4%, 31.2%, and 32.1% for PAMC26660, and 22.8%, 22.7%, and 22.9% for PAMC28562 and those of three closely related sp. strains, *V. boronicumulans* NBRC 103145^T^, *V. beijingensis* 502^T^, and *V. paradoxus* NBRC 15149^T^, respectively (Table 1). These values are lower than the 70% threshold required to categorize species. Therefore, we have checked 16S rRNA from the extended TYGS gene analysis (Supplementary Figure 1), which includes type strains. This data reveals that among our three strains (PAMC28711, PAMC26660, and PAMC28562), PAMC26660 clusters the different branches. ANI tests of PAMC28711 revealed 84.24%, 79.97%, and 80.45% (ANIb) and 85.61%, 85.51%, and 85.56% (ANIm) relatedness with *V. paradoxus* NBRC15149^T^, *V. beijingensis* 502^T^, and *V. brononicumulans* NBRC103145^T^, respectively. Likewise, ANI tests of PAMC26660 revealed 84.24%, 84.17%, and 85.19% (ANIb) and 88.01%, 87.95, and 88.5% (ANIm) relatedness with *V. paradoxus* NBRC15149^T^, *V. beijingensis* 502^T^, and *V. brononicumulans* NBRC103145^T^, respectively. ANI tests of PAMC28562 revealed 78.77%, 78.59%, and 78.62% (ANIb) and 84.96%, 84.88%, and 84.85% (ANIm) relatedness with *V. paradoxus* NBRC15149^T^, *V. beijingensis* 502^T^, and *V. brononicumulans* NBRC103145^T^, respectively (Tables 2 and S6). As a result, all the outcomes were significantly below the required sp. determination thresholds [38, 39].

### 3.3. Analysis of Trehalose Producing and Degrading CAZyme Families and Subfamilies in the Genome of *Variovorax*

Based on the CAZyme annotation outcomes, *Variovorax* sp. PAMC28711 encodes 23 GHs, 30 GTs, 2 CEs, 6 CBMSs, and 2 AAs. Similarly, strain PAMC26660 encodes 27 GHs, 43 GTs, 4 CEs, and 10 CBMs. Strain PAMC28562 encodes 27 GHs, 50 GTs, 3 CEs, and 11 CBMs. Among these three strains, PAMC28562 encodes more GTs and CBMs as compared to the other two strains, whereas PAMC28711 is the only strain that encodes AAs. Based on the CAZyme results, many related genes that synthesize and degrade enzymes of trehalose were annotated, and Table 2 and Tables S2–S5 of the Supplementary Information (SI) summarize the detailed results. The CAZyme annotation of strains identified 4 trehalose synthase enzymes: Trehalose 6-phosphate synthase (GT20); Trehalose 6-phosphate phosphatase (GT20); maltooligosyl-trehalose synthase (CBM48 and GH13); maltooligosyl-trehalose trehaldohydrolase (CBM48 and GH13); and 1 trehalose degradation enzyme: trehalase (GH15 and GH37) (Tables 3 and 4).

**Figure 2:**
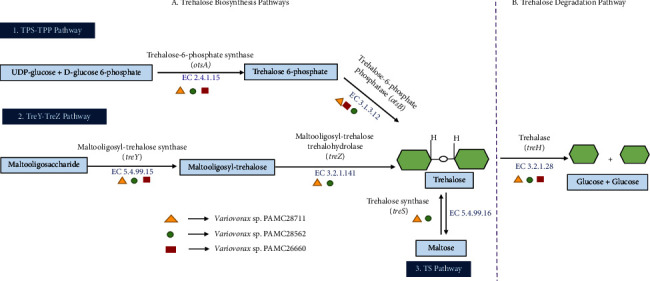
The trehalose synthesis (TPS/TPP, TS, and TreY/TreZ) pathways and degradation (TreH) pathways and their related enzymes are involved in the complete genome of Antarctica isolates (*Variovorax* sp. strains PAMC28711, PAMC26660, and PAMC28562).

**Figure 3:**
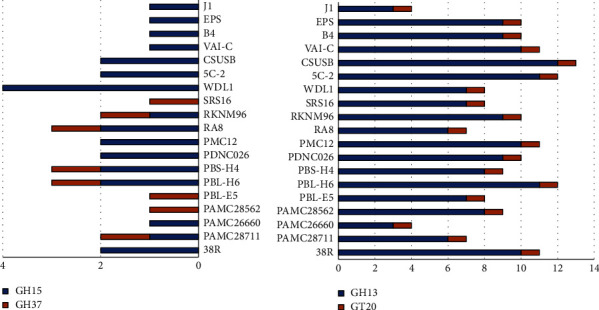
comparative analysis of trehalose synthesis and trehalose degradation enzymes based on CAZyme families found in 19 complete genomes of *Variovorax*.

On the basis of the prediction of trehalose metabolic pathways in *Variovorax* sp., our two strains PAMC28711 and PAMC28562 encode two TPS/TPP (GT20/GT21) (enzymes such as trehalose phosphate synthase and trehalose 6-phosphate phosphatase) pathway enzymes and two TreY/TreZ (CBM48 and GH13/CBM48 and GH14) (enzymes such as maltooligosyl-trehalose synthase and maltooligosyl-trehalose trehaldohydrolase) pathway enzymes, TS (GT13) (trehalose synthase) pathway enzyme, whereas only strain PAMC26660 encodes only two TPS/TPP (enzymes such as trehalose phosphate synthase and trehalose 6-phosphate phosphatase) pathway (Figure 2). All three strains analyzed, PAMC28711, PAMC26660, and PAMC28562, as well as other complete genomes of *Variovorax* strains that were studied for comparison, possessed various CAZyme subfamilies. Interestingly, strain PAMC28711 encodes two trehalase enzymes (GH15 and GH37) according to Prokka annotation prediction, whose COG 3387 and COG 1626 were predicted, while strain PAMC28562 encodes only one trehalase (GH37), whose COG 1626 was predicted. Likewise, strain PAMC26660 encodes only one trehlase (GH15), whose COG 3387 was predicted. None of the studied strains, along with the compared strains, encode the other two TreP (trehalose phosphorylase) pathway and TreT (trehalose glycosyl-transferring synthase) pathway enzymes (Figure 3). Tables 3 and S5 of the SI summarize the detailed annotation results obtained from using dbCAN2 (https://bcb.unl.edu/dbCAN2/) and the Prokka annotation program.

**Figure 4:**
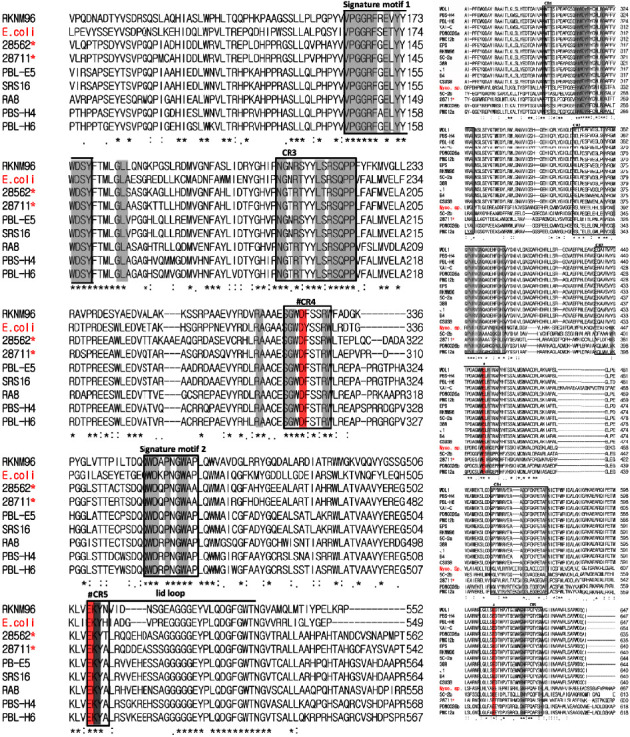
(a) Sequence alignments of the characterized GH37 trehalase and putative trehalase. “^#^” represents the putative catalytic residues, which are highlighted in red. Lid loops are indicated by thin lines. The boxes show the trehalase signature motifs 1 and 2 and the purposed GH37 conserved regions (CR3-CR5). “^*∗*^” denotes our strains that possess GH37 trehalase. Red text denotes the reference strains for comparison. (b) Sequence alignments of the characterized GH15 trehalase and putative trehalase. “^#^” symbol denotes the putative catalytic residues, which are highlighted (red). Boxes show the proposed GH15 conserved regions (CR1–CR5). “^*∗*^” denotes our strains that possess GH15 trehalase.

Based on the signature motifs analyzed through multiple sequence alignment (https://www.ebi.ac.uk/Tools/msa/clustalo/), almost all the studied strains might possess GH15 trehalose except *Variovorax* sp. PAMC28562, *Variovorax* sp. SRS16, and *Variovorax* sp. PBL-E5, as they possess GH37 trehalase only. Signature motifs, as well as other motifs, have been revealed in some *Variovorax* strains by using reference sequence *E. coli* str. K-12 substr. MG1655 (AAC76544.1), when multiple sequence alignment was performed among the strains that possessed trehalase (GH37) genes (Figure 4(a)). The *Variovorax* sp. PAMC28711, along with other *Variovorax* strains RKNM96, RA8, PSB-H6, PMC12, PDNC026, and *V. paradoxus* 5C-2, might possess both GH37 (Figure 4(a)) and GH15 trehalases (Figure 4(b)).

### 3.4. AZCL Screening of the Polysaccharide Degradation Potential of Selected Strain

The polysaccharide degradation activity was determined via AZCL screening in a selected strain PAMC28711 of an Antarctic isolate. Strain PAMC28711 degraded various polysaccharides, such as starch (AZCL-amylose), cellulose (AZCL-barley *β*-glucan and AZCL-HE-cellulose), and hemicellulose (AZCL-xylan (beech wood), AZCL-xylan (birch wood), AZCL-arabinoxylan, and AZCL-xyloglucan), computed on four different media, of Bennett's media (B's), Marine agar (MA), Malt Yeast (MY) media, and Reasoner's 2A agar (R2A) at different temperature ranges, i.e., from psychrophilic to mesophilic, as summarized in Table 5. According to the findings, starch substrates, such as AZCL-amylose degrading activity, were seen at (4, 15, and 25) °C in MA and R2A media. But no activity was observed at 37°C (Table 5). The GH13, GH15, and GH37 genes (Figure 3), in the strain *Variovorax* sp. PAMC28711, can degrade starch and other carbohydrates like trehalose.

## 4. Discussion

The study of the trehalose metabolic pathway in bacteria has attracted researchers' attention since trehalose has a wide range of industrial and therapeutic applications. It has also been observed that trehalose accumulation or production in bacteria demonstrates stress resistance to desiccation, osmotic stress, and other factors. When we compared the genomic size and GC (percent) content of our three isolates of *Variovorax* sp. PAMC28711, PAMC26660, and PAMC28562, we discovered that both strains PAMC28711 (4.32 Mb genome size; GC = 63.70 percent) and PAMC28562 (4.69 Mb genome; GC = 66 percent) have smaller genome sizes and GC content than that of the strain PAMC26660, as well as within all the *Variovorax* genomes studied here. According to Almpanis et al., there may be a correlation between chromosomal length and genome GC content. The longer the genome, the higher the GC content, which may be true for our two strains, PAMC28711 and PAMC28562, but not for one of our strains, PAMC26660, or most of the other *Variovorax* strains studied here. Furthermore, as revealed by the findings of the linear regression model, this alone is not sufficient to explain the whole variation in genome G + C content. As a result, other factors must be explored in order to explain the G + C content [40]. The organism's normal optimum temperature range is probably the most noticeable of these [41, 42]. Based on ANI values, our three strains, PAMC28711, PAMC26660, and PAMC28562, have less than 95% identity whose value did not match the sp. delineation threshold. The average nucleotide identity (ANI) is a genome similarity metric that may be applied to prokaryotic organisms regardless of their G + C composition, and a cutoff value of >95% indicates that they belong to the same sp. [39, 43]. Because orthologous genes can differ widely between genomes, ANI values do not imply genome evolution. On the other hand, ANI closely replicates the classic microbiological idea of DNA-DNA hybridization relatedness for defining sp., which is why many researchers prefer it because it considers the fluid nature of the bacterial gene pool and hence indirectly considers shared functions [33].

One of the earliest examples is the discovery that elevating trehalose levels in *Streptomyces griseus* spores increases resistance to heat and desiccation stress [44], which likely adds to actinomycete spores' capacity to endure harsh environmental conditions. The germination of spores was similarly delayed by high levels of accumulated trehalose; however, the relevance of this is unclear. Trehalose has since been revealed to protect bacterial vegetative cells from a range of abiotic stresses. *Variovorax* sp. strains PAMC28711, PAMC26660, and PAMC28562 have trehalose biosynthesis and degrading genes that might be helpful to these organisms to survive in harsh environments like Antarctica.

Generally, five distinct trehalose synthetic pathways exist in bacteria. They include the TPS/TPP pathway (enzymes such as trehalose phosphate synthase and trehalose 6-phosphate phosphatase), TS pathway (trehalose synthase), TreY/TreZ pathway (enzymes such as maltooligosyl-trehalose synthase and maltooligosyl-trehalose trehaldohydrolase), TreP pathway (trehalose phosphorylase), and TreT pathway (trehalose glycosyl-transferring synthase) [45, 46]. Among our three Antarctica isolates, two strains, PAMC28711, and PAMC28562, possess three trehalose biosynthesis pathways (TPP/TPS, TreY/TreZ, and TS), whereas PAMC26660 possesses only one biosynthesis pathway (TPP/TPS) (Table 4). As it was reported in the previous paper, the most common is the TPS/TPP biosynthesis pathway of trehalose found in bacteria. The enzymes involved in the TPS/TPP pathways include trehalose 6-phosphate and trehalose 6-phosphate phosphatase. Additionally, the TS pathway comprises a trehalose synthase enzyme that belongs to the GH13 CAZyme subfamily. This TS pathway is reversible and includes both the biosynthesis and degradation of trehalose from maltose (Table 4) [47].

Trehalase (glycoside hydrolase: EC 3.2.1.28) breaks down trehalose to produce two molecules of glucose. There are several alternative pathways for the degradation of trehalose [48]. Interestingly, bacterial trehalase is not as widely distributed as the trehalose biosynthetic pathway, since trehalose-6-phosphate synthases/phosphatases (TPSs/TPPs) occur in diverse living forms, ranging from micro-to macro-organisms [49]. The enzymes involved in trehalose degradation include *α*, *α*-trehalose phosphorylase (EC 2.4.1.64) and *α*, *α*-trehalase (EC 3.2.1.28). *E. coli* strain K12 contains two trehalases (cytoplasmic trehalase, TreF and periplasmic trehalase, TreA) [50]. TreF was predicted via the KEGG pathway map and CAZyme database in *Variovorax* sp. strains PAMC28711 and PAMC28562. TreF is the enzyme responsible for the degradation of the disaccharide *α*, *α*-trehalose, yielding two glucose subunits [51]. In the carbohydrate-active enzymes (https://www.cazy.org/) database, trehalases were originally classified into glycoside hydrolase 37 (GH37), 15 (GH15), and 65 (GH65). GH37 is a CAZyme subfamily that is comprised of only one enzyme, trehalase, but some acid trehalases and some phosphorylases belong to the GH65 CAZyme subfamily. In *Mycobacterium smegmatis* and *M. tuberculosis,* GH15 trehalase was discovered and reported in 2007 [52]. The catalytic domains (CDs) with (*α*/*α*)_6_-barrel architecture are found in GH37, GH65, and GH15 enzymes. GH37 has two catalytic residues, Asp and Glu, whereas GH65 and GH15 have Asp and Glu residues, which might be involved in the common inverting catalytic mechanism [53]. In their fundamental structures, GH37 enzymes, two well-known trehalase signature motifs were found, motif 1 (PGGRFXEXY[G/Y] WD[S/T] Y) and motif 2 (QWD[Y/F] P[N/Y][G/A]W[P/A]P), whereas GH65 and GH15 trehalases lack these motifs [49, 51]. In the CDs of GH37 enzymes, in addition to the two well-known trehalase signature motifs 1 and 2, three CRs (motifs) are also suggested: motifs 3 (N[A/G] XRXYYXXRSQPP), 4 (SGXD[T/F] [S/T] [S/T/Y] R[F/L/W]), and 5 (EK[Y/F] D). The two catalytic residues stated above are found in Motifs 4 and 5. Lip loop regions are also observed in motif 5, which may play an important role in substrate recognition [54]. A cytoplasmic trehalase was found using the CAZyme database, based on the results of rapid annotations using subsystems technology (RAST) annotation [17].

Among the three Antarctic isolates studied, only strain PAMC28711 has both trehaloses GH37 and GH15, which are found in a small number of other *Variovorax* strains as well. *Mycrolicibacterium smegmatis* MC2155 was used as a reference sequence (ABK72415.1). The signature motif of GH15 trehalase differs from that of GH37 trehalase. GAs and glucodextranases (GDases) are GH15 enzymes that have five CRs in their basic structures, which are assumed to represent the active sites. In hydrolytic reactions, two Glu residues in GH15 CRs 3 and 5 are important [55–58]. WE[F/D/E/V] and [S/G/A] E[E/H] are analogous regions in GH15 trehalose, where a comparison of GA sites, WEE and [S/P/N] EQ, revealed two Glu residues at identical positions to be significant for the catalytic process as catalytic residues. It has been reported that trehalases from the GH15 family have greater *K*_*M*_ values for trehalase than trehalases from other families [59].

According to the CAZyme database, GH15 genes encode for *α*, *α*-trehalase, as well as additional genes, including starch glucoamylase, glucodextranase, and dextran dextrinase, whereas GH37 genes encode for single *α*, *α*-trehalase, indicating the presence of trehalose catabolism in the cell [13].

The degradation activity of cellulose substrates, such as AZCL-HE-cellulose and AZCL-barley-*β*-glucan, was determined, with AZCL-barley-glucan showing degradation activity throughout all temperature ranges in B's media. In addition, MA and R2A media, the substrate AZCL-HE-cellulose, demonstrated degrading activity at temperatures of 15 and 25°C. Most cellulolytic enzymes are hydrolases (glycoside hydrolases or GHs), a subclass of carbohydrate-active enzymes (CAZymes). Cellulolytic enzymes are mostly found in the GH1, GH3, GH5, GH6, GH7, GH8, GH9, GH12, GH45, and GH48 families [45]. In hemicellulose substrates, such as AZCL-arabinoxylan, AZCL-xylan (Beech wood), AZCL-xylan (birch wood), and AZCL-xyloglucan degradation activity, strain PAMC28711 showed hemicellulose degradation activity in AZCL-arabinoxylan with all the temperatures provided in B's, MA, and R2A media, apart from MY media, which showed activity only at 25°C. In the temperature range of 4–37°C, the substrate AZCL-xylan (beech wood) demonstrated hemicellulose breakdown. Hemicellulolytic enzymes are classified as members of the GH2, GH10, GH11, GH16, GH26, GH30, GH31, GH39, GH42, GH43, and GH53 families in the CAZyme database [46]. Hemicellulose, like cellulose, has considerable potential in terms of bioenergy and biotechnological applications. GH2, GH10, GH11, GH16, GH26, GH30, GH31, GH36, GH43, GH51, GH74, and GH95 are common hemicellulolytic enzymes. Importantly, members of the same GH family may catalyze distinct processes, and their family membership may not be enough to determine their activity's targets [46]. In addition, strain PAMC28711 showed the ability to degrade AZCL substrates mainly at the mesophilic temperature, i.e., at 25°C. This screening method has been utilized in several studies [60, 61], which somewhat confirms its reliability.

## 5. Conclusions

In summary, the complete genomes of *Variovorax* strains PAMC28711, PAMC28562, and PAMC26660 were compared with the complete genomes of *Variovorax* that were deposited at the NCBI until October 2021. A comparative analysis of the obtained genome showed that strain PAMC26660 has only one complete trehalose biosynthesis pathway (TPS/TPP), whereas strains PAMC28711 and PAMC28562 possess all three complete trehalose biosynthesis pathways (TPS/TPP, TS, and TreY/TreZ). In addition, it was found that only strain PAMC28711 has two trehalases (GH37 and GH15) among the three Antarctica isolates studied here. Based on the results of AZCL screening, the strain PAMC28711 thrived at 25°C even though it was isolated from cold-adapted lichen. Based on 16S rRNA sequence analysis and ANI value similarity with other *Variovorax* sp., the two isolates, PAMC28562 and PAMC26660, have been confirmed as *Variovorax* sp. There have been no previous studies of the trehalose metabolic pathway in *Variovorax*, including isolates from Antarctica. Strains PAMC28711, PAMC28562, and PAMC26660 are anticipated to be able to synthesize and degrade trehalose. Furthermore, a genomic comparison of *Variovorax* sp. along with Antarctica isolates demonstrated that these cold-adapted organisms can withstand harsh environments. In conclusion, we expect the genome sequence analysis might provide additional information regarding the role of trehalose biosynthesis and degrading encoding genes that are active at low temperatures and can be employed for biotechnological applications and fundamental research purposes.

## Figures and Tables

**Table 1:** Genomic information and comparative data on trehalose metabolic pathways of 19 strains of *Variovorax*, including our three strains PAMC26660, PAMC28562, and PAMC28711, which are denoted by red asterisk.

Strains			*Variovorax* sp.													*Variovorax paradoxus*					*Variovorax boronicumulans*
			PAMC 26660^∗^	RKNM 96	38R	WDL1	RA8	PBS-H4	PBL-H6	PMC 12	PDNC 026	SRS 16	PBL-E5	PAMC 28562^∗^	PAMC 28711^∗^	5C-2	CSUSB	EPS	B4	VAI-C	J1
Genome information		Size (Mb)	7.39	7.17	6.87	8.36	7.42	6.65	6.87	7.02	7.11	7.67	7.09	4.69	4.32	7.29	5.57	6.55	7.15	9.44	7.14
		GC%	66.00	66.70	67.50	66.38	66.84	66.80	66.24	67.62	67.57	66.52	66.68	63.70	66.00	67.30	65.70	66.50	67.14	69.06	68.00
		CDS	6801	6592	6290	7862	6904	6186	6386	6458	6481	7281	6799	4298	4071	6653	5138	5981	6678	8568	6443
		Genes	6919	6700	6405	8177	7151	6333	6595	6582	6613	7534	7012	4402	4232	6811	5236	6097	6831	8763	6727
		tRNAs	52	58	51	119	74	50	95	57	56	97	95	47	46	52	52	57	48	52	51
Trehalose metabolism genes	Synthesis	*otsA*	√	√	√	√	√	√	√	√	√	√	√	√	√	√	√	√	√	√	√
		*otsB*	√	√	√	√	√	√	√	√	√	√	√	√	√	√	√	√	√	√	√
		*treY*	√	√	√	√	√	√	√	√	√	√	√	√	√	√	√	√	√	√	*X*
		*treZ*	*X*	√	√	√	√	√	√	√	√	√	√	√	√	√	√	√	√	√	*X*
		*treS*	*X*	√	√	√	√	√	√	√	√	√	√	√	√	√	√	√	√	√	*X*
	Degradation	*treH*	GH15	GH15	GH15	GH15	GH15	GH15	GH15	GH15	GH15	*X*	*X*	*X*	GH15	GH15	GH15	GH15	GH15	GH15	GH15
			*X*	GH37	*X*	*X*	GH37	GH37	GH37	*X*	GH37	GH37	GH37	GH37	GH37	*X*	*X*	*X*	*X*	*X*	*X*
		*treP*	*X*	*X*	*X*	*X*	*X*	*X*	*X*	*X*	*X*	*X*	*X*	*X*	*X*	*X*	*X*	*X*	*X*	*X*	*X*
		*treT*	*X*	*X*	*X*	*X*	*X*	*X*	*X*	*X*	*X*	*X*	*X*	*X*	*X*	*X*	*X*	*X*	*X*	*X*	*X*

√: present reference to dbCAN, Prokka anotation, and GenBank locus tag. GH15: availability reference to glycoside hydrolase 15 (GH15) family. GH37: availability reference to glycoside hydrolase 37 (GH37) family. *X*: not present.

**Table 2:** Average nucleotide identity and digital DNA-DNA hybridization values between strains of *Variovorax* sp. PAMC28711, PAMC28562, and PAMC2660 and their related species.

Strain/analysis	Query strains	Type strains		
		*Variovorax paradoxus* NBRC15149^T^	*Variovorax beijingensis* 502^T^	*Variovorax boronicumulans* NBRC103145^T^
ANIb (%)	*Variovorax* sp. PAMC28711	84.24	79.97	80.45
ANIm (%)		85.61	85.51	85.56
dDDH (d4, %)		24.5	24.3	24.5

ANIb (%)	*Variovorax* sp. PAMC28562	78.77	78.59	78.62
ANIm (%)		84.96	84.88	84.85
dDDH (d4, %)		22.8	22.7	22.9

ANIb (%)	*Variovorax* sp. PAMC26660	84.24	84.17	85.19
ANIm (%)		88.01	87.95	88.5
dDDH (d4, %)		31.4	31.2	32.1

**Table 3:** Comparative analysis of the number of putative genes for the six CAZyme categories in the selected complete genomes of *Variovorax* strains.

Species	GH^a^	GT^b^	PL^c^	CE^d^	CBM^e^	AA^f^	Total
*Variovorax* sp. 38R	34	45	0	4	12	0	95
*Variovorax* sp. PAMC28711^*∗*^	23	30	0	3	6	2	64
*Variovorax* sp. PAMC26660^*∗*^	27	43	0	4	10	0	84
*Variovorax* sp. PAMC28562^*∗*^	27	50	0	3	11	0	91
*Variovorax* sp. PBL-E5	26	52	0	3	9	0	90
*Variovorax* sp. PBL-H6	34	44	0	3	9	0	90
*Variovorax* sp. PBS-H4	33	44	0	4	9	0	90
*Variovorax* sp. PDNC026	32	41	0	3	13	0	89
*Variovorax* sp. PMC12	34	42	0	4	13	0	93
*Variovorax* sp. RA8	29	53	0	4	9	0	95
*Variovorax* sp. RKNM96	31	50	0	4	13	0	98
*Variovorax* sp. SRS16	29	48	0	4	9	0	90
*Variovorax* sp. WDL1	41	53	0	3	9	0	106
*Variovorax paradoxus* 5C-2	34	38	0	4	13	1	90
*Variovorax paradoxus* CSUSB	35	36	1	3	12	0	87
*Variovorax paradoxus* VAI-C	36	46	0	7	13	0	102
*Variovorax paradoxus* B4	31	47	0	5	10	0	93
*Variovorax paradoxus* EPS	31	46	1	3	11	0	92
*Variovorax boronicumulans* J1	23	37	0	4	10	0	74

^a^GH: glycoside hydrolases; ^b^GT: glycosyl transferase; ^c^PL: polysaccharide lyases. ^d^CE: carbohydrate esterases; ^e^CBM: carbohydrate-binding modules; ^f^AA: auxiliary activities.

**Table 4:** CAZyme families/subfamilies associated with trehalose metabolic pathway and their respective EC numbers.

Pathways	Genes	Gene products	EC number	CAZyme families/subfamilies
Biosynthesis	TPS	Trehalose 6-phosphate synthase	2.4.1.15	GT20
	TPP	Trehalose 6-phosphate phosphatase	3.1.3.12	GT21
	TS	Trehalose synthase	5.499.16	GH13
	TreY	Maltooligosyl-trehalose synthase	5.499.15	CBM48 and GH13
	TreZ	Maltooligosyl-trehalose trehaldohydrolase	3.2.1.141	CBM48 and GH14

Degradation	TreH	Trehalase	3.2.1.28	GH37 and GH15

**Table 5:** AZCL screening for the polysaccharide degradation potential of *Variovorax* sp. PAMC28711. The screening involves the analysis of cellulose, hemicellulose, and starch degradation abilities.

	AZCL substrates																							
	AZCL-amylose				AZCL-barley *β*-glucan				AZCL-arabinoxylan				AZCL-HE-cellulose				AZCL-xylan (beech wood)				AZCL-xylan (birch wood)			
Media/temp (°C)	**4**	**15**	**25**	**37**	**4**	**15**	**25**	**37**	**4**	**15**	**25**	**37**	**4**	**15**	**25**	**37**	**4**	**15**	**25**	**37**	**4**	**15**	**25**	**37**
B's	−	−	+	−	+	+	+	+	+	+	+	+	−	−	+	−	−	−	+	+	−	−	+	+
MA	+	+	+	−	−	+	+	+	+	+	+	+	−	−	+	−	+	+	+	+	+	+	+	+
MY	−	−	+	−	−	−	+	−	−	−	+	−	−	−	+	−	−	−	+	−	−	+	+	−
R2A	+	+	+	−	−	+	+	+	+	+	+	+	−	+	+	−	+	+	+	−	+	+	+	−

## Data Availability

On reasonable request, the corresponding author will provide the datasets used and analyzed during the current study.

## Abbreviations

AA: Auxiliary activity
ANI: Average nucleotide identity
AZCL: Azurine cross-linked
B's: Bennett's agar
CAZyme: Carbohydrate-active enzyme
CBM: Carbohydrate-binding module
CE: Carbohydrate esterase
GH: Glycoside hydrolase
GT: Glycosyltransferase
KEGG: Kyoto encyclopedia of genes and genomics
MY: Malt yeast media
MA: Marine agar
NCBI: National center for biotechnology information
PL: Polysaccharide lyase
R2A: Reasoner's 2A agar
SI: Supplementary information
VFDB: Virulence factor database.

## Supplementary Materials

Supplementary Materials Supplementary Table S1: genomic information of the three complete metagenomic assembled genomes of *Variovorax* sp. Supplementary Table S2: comparison of glycoside hydrolases (GHs) families and subfamilies among the complete genome of *Variovorax* strains. Supplementary Table S3: comparison of glycosyl transferases (GTs) families and subfamilies among the complete genome of *Variovorax* strains. Supplementary Table S4: comparison of carbohydrate esterases (CEs), carbohydrate-binding modules (CBMs), auxiliary activities (AAs), and polysaccharide lyases (PLs) families and subfamilies among the complete genome of *Variovorax* strains. Supplementary Table S5: the CAZyme subfamilies of trehalose metabolism of the complete *Variovorax* strains by dbCAN2 meta server, Prokka annotation, and GenBank locus tag. Supplementary Table S6: pairwise comparisons of query strains vs. type strain genomes. Supplementary Figure 1: ML (Maximum Likelihood) tree of the *Variovorax* dataset inferred under the GTR + GAMMA model. The tree is rooted at the midpoint, and branches are scaled in terms of the expected number of substitutions per site. The numbers above the branches are support values when larger than 60% from ML (left) and MP (right) bootstrapping. The tree was inferred through TYGS via the DSMZ gene phylogeny pipeline available under https://ggdc.dsmz.de/phylogeny-service.php.
