# Supplementary material for: Complete Genome Sequence and Comparative Genome Analysis of Variovorax sp. Strains PAMC28711, PAMC26660, and PAMC28562 and Trehalose Metabolic Pathways in Antarctica Isolates
Source: Int J Microbiol. 2022 Nov 9;2022:5067074. doi: 10.1155/2022/5067074 (PMC10232917; doi:10.1155/2022/5067074)

**Complete genome sequence and comparative genome analysis of *Variovorax* sp. strains PAMC28711, PAMC26660, and PAMC28562, trehalose metabolic pathways in Antarctica isolates**

Prasansah Shrestha^1^, Jayram Karmacharya^1^, So-Ra Han^1^, Jun Hyuck Lee^2,3^, Hyun Park^4^, and Tae-Jin Oh^1,4,5, *^

^1^ Department of Life Science and Biochemical Engineering, Graduate School, Sun Moon University, Asan 31460, Korea.

^2^ Unit of Research for Practical Application, Korea Polar Research Institute, Incheon 21990, Korea.

^3^ Department of Polar Sciences, University of Science and Technology, Incheon 21990, Korea

^4^ Division of Biotechnology, College of Life Sciences and Biotechnology, Korea University, Seoul 02841, Korea.

^5^ Genome-based Bio IT Convergence Institute, Asan 31460, Korea.

^6^ Department of Pharmaceutical Engineering and Biotechnology, Sun Moon University, Asan 31460, Korea.

Corresponding author: Prof. T.-J. Oh

Department of Pharmaceutical Engineering and Biotechnology, Sun Moon University, Asan 31460, Korea. Tel.: +82 41 530 2677; E-mail: [tjoh3782@sunmoon.ac.kr](mailto:tjoh3782@sunmoon.ac.kr)

| **Feature** | **PAMC28711** | **PAMC26660** | **PAMC28562** |
| --- | --- | --- | --- |
| **A. Genome Statistics** |  |  |  |
| Contigs | 1 | 1 | 1 |
| Total length bp | 4316152 | 7388698 | 4693528 |
| N50 | 4 | 4 | 7 |
| L50 | 1 | 1 | 1 |
| GC% | 66 | 66 | 63.7 |
| **B. Genome Features** |  |  |  |
| Biosample | [SAMN04457487](https://www.ncbi.nlm.nih.gov/biosample/SAMN04457487) | [SAMN15794718](https://www.ncbi.nlm.nih.gov/biosample/SAMN15794718) | [SAMN15794725](https://www.ncbi.nlm.nih.gov/biosample/SAMN15794725) |
| Chromosome genes | 4232 | 6919 | 4402 |
| Protein-coding genes | 4071 | 6801 | 4298 |
| Pseudogenes | 106 | 57 | 48 |
| rRNA genes | 6 | 6 | 6 |
| tRNA genes | 46 | 52 | 47 |
| Genome quality | Good | Good | Good |
| Coarse consistency | 98.1 | 98.2 | 97.3 |
| Fine Consistency | 95 | 94.3 | 95.6 |
| CheckM contamination | 3.5 | 0 | 0 |

**Supplementary Table S1.** Genomic information of the three complete metagenomic assembled genomes of *Variovorax* sp.

**Supplementary Table S2.** Comparison of Glycoside Hydrolases (GHs) families and subfamilies among the complete genome of *Variovorax* strains.

| **GH** | | | | | | | | | | | | | | | | | | | | | | | | | | | |
| --- | --- | --- | --- | --- | --- | --- | --- | --- | --- | --- | --- | --- | --- | --- | --- | --- | --- | --- | --- | --- | --- | --- | --- | --- | --- | --- | --- |
| **Genus** | **GH1** | **GH3** | **GH8** | **GH10** | **GH13** | **GH15** | **GH16** | **GH19** | **GH23** | **GH24** | **GH37** | **GH39** | **GH43** | **GH51** | **GH73** | **GH77** | **GH92** | **GH94** | **GH95** | **GH102** | **GH103** | **GH104** | **GH105** | **GH108** | **GH144** | **GH153** | **NC** |
| ***V.* sp*.* 38R** | 0 | 4 | 1 | 0 | 10 | 2 | 0 | 0 | 5 | 2 | 0 | 0 | 0 | 0 | 1 | 1 | 0 | 1 | 1 | 2 | 1 | 0 | 0 | 0 | 1 | 1 | 1 |
| ***V.* sp. PAMC28711*** | 0 | 2 | 0 | 0 | 6 | 1 | 0 | 0 | 5 | 1 | 1 | 0 | 0 | 0 | 1 | 0 | 0 | 1 | 0 | 2 | 2 | 0 | 0 | 1 | 0 | 0 | 0 |
| ***V.* sp. PAMC26660*** | 0 | 3 | 0 | 0 | 3 | 1 | 0 | 1 | 6 | 3 | 0 | 0 | 0 | 0 | 1 | 0 | 2 | 1 | 0 | 2 | 1 | 2 | 0 | 0 | 0 | 1 | 0 |
| ***V.* sp. PAMC28562*** | 0 | 2 | 1 | 0 | 8 | 0 | 0 | 0 | 6 | 0 | 1 | 1 | 0 | 1 | 1 | 1 | 0 | 1 | 0 | 2 | 2 | 0 | 0 | 0 | 0 | 0 | 0 |
| ***V.*sp. PBL-E5** | 0 | 2 | 0 | 0 | 7 | 0 | 0 | 1 | 8 | 0 | 1 | 0 | 0 | 0 | 1 | 1 | 0 | 1 | 0 | 2 | 2 | 0 | 0 | 0 | 0 | 0 | 0 |
| ***V.*sp*.* PBL-H6** | 0 | 2 | 0 | 0 | 11 | 2 | 0 | 1 | 8 | 0 | 1 | 0 | 0 | 0 | 1 | 1 | 0 | 0 | 0 | 1 | 1 | 0 | 1 | 0 | 1 | 0 | 3 |
| ***V.* sp. PBS-H4** | 0 | 2 | 1 | 0 | 8 | 2 | 0 | 1 | 9 | 2 | 1 | 0 | 0 | 0 | 1 | 1 | 0 | 0 | 0 | 1 | 1 | 0 | 0 | 0 | 1 | 0 | 2 |
| ***V.* sp. PDNC026** | 0 | 3 | 1 | 0 | 9 | 2 | 1 | 0 | 5 | 2 | 0 | 0 | 0 | 0 | 1 | 1 | 0 | 1 | 0 | 1 | 1 | 0 | 0 | 0 | 0 | 1 | 2 |
| ***V.* sp. PMC12** | 0 | 3 | 1 | 0 | 10 | 2 | 1 | 1 | 6 | 1 | 0 | 0 | 0 | 0 | 1 | 1 | 0 | 1 | 0 | 2 | 1 | 0 | 0 | 0 | 0 | 1 | 1 |
| ***V.* sp. RA8** | 0 | 2 | 1 | 0 | 6 | 2 | 0 | 1 | 7 | 0 | 1 | 0 | 0 | 0 | 1 | 1 | 0 | 1 | 0 | 2 | 1 | 0 | 0 | 1 | 0 | 0 | 1 |
| ***V.* sp. RKNM96** | 0 | 3 | 1 | 0 | 9 | 1 | 0 | 0 | 6 | 0 | 1 | 0 | 0 | 0 | 0 | 1 | 1 | 1 | 1 | 2 | 1 | 0 | 0 | 0 | 0 | 1 | 2 |
| ***V.* sp. SRS16** | 0 | 2 | 0 | 0 | 7 | 0 | 0 | 1 | 9 | 0 | 1 | 0 | 0 | 0 | 2 | 1 | 0 | 1 | 0 | 2 | 2 | 1 | 0 | 0 | 0 | 0 | 0 |
| ***V.* sp*.* WDL1** | 0 | 3 | 0 | 0 | 7 | 4 | 0 | 2 | 12 | 0 | 0 | 0 | 0 | 0 | 1 | 1 | 0 | 2 | 0 | 2 | 1 | 0 | 1 | 0 | 3 | 0 | 2 |
| ***V. paradoxus 5*C-2** | 0 | 3 | 1 | 1 | 11 | 2 | 0 | 0 | 5 | 2 | 0 | 0 | 0 | 0 | 1 | 1 | 0 | 0 | 0 | 2 | 1 | 0 | 0 | 0 | 0 | 1 | 3 |
| ***V. paradoxus* CSUSB** | 1 | 3 | 0 | 0 | 12 | 2 | 0 | 1 | 5 | 0 | 0 | 0 | 0 | 0 | 1 | 0 | 0 |  | 0 | 2 | 2 | 0 | 0 | 0 | 1 | 0 | 4 |
| ***V. paradoxus* VAI-C** | 0 | 3 | 1 | 0 | 10 | 1 | 0 | 3 | 6 | 2 | 0 | 0 | 0 | 0 | 1 | 1 | 0 | 1 | 0 | 3 | 1 | 0 | 0 | 0 | 0 | 1 | 2 |
| ***V. paradoxus* B4** | 0 | 2 | 0 | 0 | 9 | 1 | 0 | 1 | 5 | 1 | 0 | 0 | 0 | 0 | 1 | 1 | 0 | 1 | 0 | 3 | 2 | 0 | 0 | 0 | 0 | 1 | 3 |
| ***V. paradoxus* EPS** | 1 | 3 | 1 | 0 | 9 | 1 | 0 | 0 | 5 | 1 | 0 | 0 | 1 | 0 | 0 | 1 | 0 | 0 | 1 | 2 | 1 | 0 | 0 | 0 | 0 | 0 | 4 |
| ***V. boronicumulans* J1** | 0 | 3 | 0 | 0 | 3 | 1 | 0 | 5 | 5 | 0 | 0 | 0 |  | 0 | 1 | 0 | 0 | 1 | 0 | 1 | 1 | 0 | 0 | 0 | 0 | 1 | 1 |

“**NC**” mean non-classified “*” represents our strains studied in this paper.

**Supplementary Table S3.** Comparison of Glycosyl Transferases (GTs) families and subfamilies among the complete genome of *Variovorax* strains.

| **Genus** | **GT1** | **GT2** | **GT4** | **GT5** | **GT11** | **GT14** | **GT17** | **GT19** | **GT20** | **GT28** | **GT30** | **GT35** | **GT41** | **GT51** | **GT83** | **GT84** | **GT87** | **GT102** | **GT104** | **NC** |
| --- | --- | --- | --- | --- | --- | --- | --- | --- | --- | --- | --- | --- | --- | --- | --- | --- | --- | --- | --- | --- |
| ***V.* sp*.* 38R** | 1 | 15 | 8 | 1 | 0 | 0 | 0 | 1 | 1 | 2 | 1 | 1 | 1 | 6 | 0 | 1 | 0 | 0 | 1 | 5 |
| ***V.* sp. PAMC28711*** | 0 | 14 | 3 | 1 | 0 | 0 | 0 | 1 | 1 | 2 | 1 | 1 | 0 | 5 | 0 | 0 | 0 | 0 | 1 | 0 |
| ***V.* sp. PAMC26660*** | 1 | 19 | 3 | 1 | 0 | 0 | 0 | 1 | 1 | 2 | 1 | 1 | 2 | 6 | 2 | 1 | 0 | 0 | 1 | 1 |
| ***V.* sp. PAMC28562*** | 1 | 20 | 13 | 1 | 0 | 0 | 1 | 1 | 1 | 2 | 1 | 1 | 0 | 5 | 0 | 1 | 0 | 0 | 1 | 1 |
| ***V.* sp. PBL-E5** | 1 | 15 | 15 | 1 | 1 | 0 | 0 | 1 | 1 | 2 | 1 | 1 | 2 | 5 | 3 | 1 | 0 | 0 | 1 | 1 |
| ***V.* sp*.* PBL-H6** | 0 | 15 | 17 | 0 | 0 | 0 | 0 | 1 | 1 | 1 | 1 | 0 | 1 | 5 | 0 | 0 | 0 | 0 | 1 | 1 |
| ***V.* sp. PBS-H4** | 0 | 15 | 12 | 0 | 0 | 0 | 0 | 1 | 1 | 2 | 1 | 1 | 0 | 5 | 2 | 0 | 0 | 0 | 1 | 3 |
| ***V.* sp. PDNC026** | 0 | 19 | 6 | 1 | 0 | 0 | 0 | 1 | 1 | 2 | 1 | 1 | 1 | 6 | 0 | 1 | 0 | 0 | 1 | 0 |
| ***V.* sp. PMC12** | 0 | 19 | 7 | 1 | 0 | 0 | 0 | 1 | 1 | 2 | 1 | 1 | 1 | 6 | 0 | 1 | 0 | 0 | 1 | 0 |
| ***V.* sp. RA8** | 0 | 16 | 19 | 0 | 0 | 0 | 0 | 1 | 1 | 2 | 1 | 1 | 1 | 1 | 1 | 1 | 1 | 0 | 1 | 2 |
| ***V.* sp. RKNM96** | 2 | 17 | 9 | 1 | 0 | 0 | 0 | 1 | 1 | 2 | 1 | 1 | 1 | 6 | 1 | 1 | 0 | 0 | 1 | 5 |
| ***V.* sp. SRS16** | 0 | 15 | 14 | 1 | 1 | 0 | 0 | 1 | 1 | 1 | 1 | 1 | 1 | 5 | 3 | 1 | 0 | 0 | 1 | 0 |
| ***V.* sp*.* WDL1** | 0 | 18 | 19 | 0 | 0 | 0 | 0 | 1 | 1 | 2 | 1 | 1 | 0 | 5 | 0 | 1 | 1 | 0 | 1 | 2 |
| ***V. paradoxus 5*C-2** | 1 | 13 | 8 | 1 | 0 | 0 | 0 | 1 | 1 | 2 | 1 | 1 | 1 | 5 | 0 | 0 | 0 | 0 | 1 | 2 |
| ***V. paradoxus* CSUSB** | 1 | 10 | 11 | 1 | 0 | 1 | 0 | 1 | 1 | 2 | 1 | 1 | 0 | 5 | 0 | 0 | 0 | 0 | 1 | 0 |
| ***V. paradoxus* VAI-C** | 2 | 17 | 8 | 1 | 0 | 0 | 1 | 1 | 1 | 2 | 0 | 1 | 1 | 6 | 0 | 1 | 0 | 1 | 1 | 2 |
| ***V. paradoxus* B4** | 2 | 18 | 11 | 1 | 0 | 0 | 0 | 1 | 1 | 2 | 1 | 1 | 1 | 4 | 1 | 1 | 0 | 0 | 1 | 0 |
| ***V. paradoxus* EPS** | 1 | 15 | 13 | 1 | 0 | 0 | 1 | 1 | 1 | 2 | 1 | 1 | 1 | 6 | 0 | 0 | 0 | 0 | 1 | 1 |
| ***V. boronicumulans* J1** | 1 | 15 | 5 | 1 | 0 | 0 | 0 | 1 | 1 | 2 | 1 | 1 | 0 | 6 | 0 | 1 | 0 | 0 | 1 | 1 |

“**NC**” mean non-classified “*” represents our strains studied in this paper.

**Supplementary Table S4.** Comparison of Carbohydrate Esterases (CEs), Carbohydrate-Binding Modules (CBMs), Auxiliary Activities (AAs), and Polysaccharide Lyases (PLs) families and subfamilies among the complete genome of *Variovorax* strains.

|  | **CE** | | **CBM** | | | | | | | **AA** | | | **PL** |
| --- | --- | --- | --- | --- | --- | --- | --- | --- | --- | --- | --- | --- | --- |
| **Genus** | **CE4** | **CE11** | **CBM12** | **CBM13** | **CBM32** | **CBM42** | **CBM48** | **CBM50** | **CBMNC** | **AA10** | **AA48** | **AA50** | **PL1** |
| ***V.* sp. 38R** | 3 | 1 | 0 | 2 | 0 | 0 | 5 | 5 | 0 | 0 | 4 | 2 | 0 |
| ***V.* sp. PAMC28711*** | 2 | 1 | 0 | 0 | 0 | 0 | 4 | 2 | 0 | 0 | 0 | 0 | 0 |
| ***V.* sp. PAMC26660*** | 3 | 1 | 0 | 1 | 0 | 0 | 2 | 7 | 0 | 0 | 0 | 0 | 0 |
| ***V.* sp. PAMC28562*** | 2 | 1 | 0 | 0 | 0 | 0 | 5 | 6 | 0 | 0 | 0 | 0 | 0 |
| ***V.* sp. PBL-E5** | 2 | 1 | 0 | 0 | 0 | 0 | 4 | 5 | 0 | 0 | 0 | 0 | 0 |
| ***V.* sp. PBL-H6** | 2 | 1 | 0 | 0 | 0 | 0 | 4 | 5 | 0 | 0 | 0 | 0 | 0 |
| ***V.* sp. PBS-H4** | 3 | 1 | 0 | 0 | 0 | 0 | 4 | 5 | 0 | 0 | 0 | 0 | 0 |
| ***V.* sp. PDNC026** | 2 | 1 | 0 | 1 | 1 | 0 | 5 | 6 | 0 | 0 | 0 | 0 | 0 |
| ***V.* sp. PMC12** | 3 | 1 | 0 | 1 | 1 | 0 | 6 | 5 | 0 | 0 | 0 | 0 | 0 |
| ***V.* sp. RA8** | 3 | 1 | 0 | 0 | 0 | 0 | 3 | 6 | 0 | 0 | 0 | 0 | 0 |
| ***V.* sp. RKNM96** | 3 | 1 | 0 | 1 | 0 | 0 | 4 | 7 | 1 | 0 | 0 | 0 | 0 |
| ***V.* sp. SRS16** | 3 | 1 | 0 | 0 | 0 | 0 | 4 | 5 | 0 | 0 | 0 | 0 | 0 |
| ***V.* sp. WDL1** | 2 | 1 | 0 |  | 0 | 0 | 3 | 6 | 0 | 0 | 0 | 0 | 0 |
| ***V. paradoxus* 5C-2** | 3 | 1 | 1 | 2 | 0 | 0 | 5 | 5 | 0 | 1 | 0 | 0 | 0 |
| ***V. paradoxus* CSUSB** | 2 | 1 | 0 | 1 | 0 | 0 | 5 | 6 | 0 | 0 | 0 | 0 | 1 |
| ***V. paradoxus* VAI-C** | 6 | 1 | 0 | 1 | 0 | 0 | 6 | 6 | 0 | 0 | 0 | 0 | 0 |
| ***V. paradoxus* B4** | 4 | 1 | 0 | 1 | 0 | 0 | 5 | 4 | 0 | 0 | 0 | 0 | 0 |
| ***V. paradoxus* EPS** | 2 | 1 | 0 | 1 | 0 | 1 | 5 | 3 | 0 | 0 | 0 | 0 | 0 |
| ***V. boronicumulans* J1** | 3 | 1 | 0 | 2 | 0 | 0 | 2 | 6 | 0 | 0 | 0 | 0 | 0 |

“**NC**” mean non-classified “*” represents our strains studied in this paper.

**Supplementary Table S5.** The CAZyme subfamilies of trehalose metabolism of the complete *Variovorax s*trains by dbCAN2 meta server, Prokka annotation and GenBank locus tag.

| **Strains** | **Gene ID** | **HMMER** | **DIAMOND** | **Hotpep** | **Prokka annotation (COG number)** | **GenBank**  **(Locus tag**) | **Functions** |
| --- | --- | --- | --- | --- | --- | --- | --- |
| ***V.* sp*.* PAMC26660*** | [NZ_CP060295.1_2894](https://bcb.unl.edu/dbCAN2/domain.php?jobid=2021101605700&gene=NZ_CP060295.1_2894) | CBM48+GH13  _11 | CBM48+GH13  __11 | GH13+GH77+  CBM48 | COG0296 |  | Malto-oligosyltrehalose  trehalohydrolase |
|  | [NZ_CP060295.1_3401](https://bcb.unl.edu/dbCAN2/domain.php?jobid=2021101605700&gene=NZ_CP060295.1_3401) | [GT20](http://www.cazy.org/GT20.html) | [GT20](http://www.cazy.org/GT20.html) | [GT20](http://www.cazy.org/GT20.html) | COG0380/  COG1877 |  | α, α-trehalose-phosphate synthase /  trehalose-6-P phosphatase |
|  | [NZ_CP060295.1_3402](https://bcb.unl.edu/dbCAN2/domain.php?jobid=2021101605700&gene=NZ_CP060295.1_3402) | [GH15](http://www.cazy.org/GH15.html) | [GH15](http://www.cazy.org/GH15.html) | [GH15](http://www.cazy.org/GH15.html) | COG3387 |  | α, α-trehalase |
| ***V.* sp. PAMC28711*** | [NZ_CP014517.1_1280](https://bcb.unl.edu/dbCAN2/domain.php?jobid=20211017221040&gene=NZ_CP014517.1_1280) | [GT20](http://www.cazy.org/GT20.html) | [GT20](http://www.cazy.org/GT20.html) | [GT20](http://www.cazy.org/GT20.html) | COG0380 |  | α, α-trehalose-phosphate synthase/  trehalose-6-P phosphatase |
|  | [NZ_CP014517.1_2051](https://bcb.unl.edu/dbCAN2/domain.php?jobid=20211017221040&gene=NZ_CP014517.1_2051) | [GH37](http://www.cazy.org/GH37.html) | [GH37](http://www.cazy.org/GH37.html) | [GH37](http://www.cazy.org/GH37.html) | COG1626 |  | α, α-trehalase |
|  | [NZ_CP014517.1_3266](https://bcb.unl.edu/dbCAN2/domain.php?jobid=20211017221040&gene=NZ_CP014517.1_3266) | GH77+GH13_26 | GH13_26+GH77 | GH13+GH77 |  | AX767_RS21965 | Malto-oligosyltrehalose synthase |
|  | [NZ_CP014517.1_3267](https://bcb.unl.edu/dbCAN2/domain.php?jobid=20211017221040&gene=NZ_CP014517.1_3267) | [GH13_10](http://www.cazy.org/GH13_10.html) | CBM48+GH13  _10 | GH13+CBM48 | COG0296 |  | Malto-oligosyltrehalose  trehalohydrolase |
|  | [NZ_CP014517.1_3269](https://bcb.unl.edu/dbCAN2/domain.php?jobid=20211017221040&gene=NZ_CP014517.1_3269) | [GH13_16](http://www.cazy.org/GH13_16.html) | [GH13_16](http://www.cazy.org/GH13_16.html) | [GH13](http://www.cazy.org/GH13.html) |  | AX767_RS16215 | Trehalose synthase |
|  | [NZ_CP014517.1_784](https://bcb.unl.edu/dbCAN2/domain.php?jobid=20211017221040&gene=NZ_CP014517.1_784) | [GH15](http://www.cazy.org/GH15.html) | [GH15](http://www.cazy.org/GH15.html) | [GH15](http://www.cazy.org/GH15.html) | COG3387 |  | α, α-trehalase |
| ***V.* sp. PAMC28562*** | [NZ_CP060296.1_1196](https://bcb.unl.edu/dbCAN2/domain.php?jobid=2021101664237&gene=NZ_CP060296.1_1196) | [GH37](http://www.cazy.org/GH37.html) | [GH37](http://www.cazy.org/GH37.html) | [GH37](http://www.cazy.org/GH37.html) | COG1626 |  | α, α-trehalase |
|  | [NZ_CP060296.1_1859](https://bcb.unl.edu/dbCAN2/domain.php?jobid=2021101664237&gene=NZ_CP060296.1_1859) | [GT20](http://www.cazy.org/GT20.html) | [GT20](http://www.cazy.org/GT20.html) | [GT20](http://www.cazy.org/GT20.html) | COG0380/  COG1877 |  | α, α-trehalose-phosphate synthase/  trehalose-6-P phosphatase |
|  | [NZ_CP060296.1_1954](https://bcb.unl.edu/dbCAN2/domain.php?jobid=2021101664237&gene=NZ_CP060296.1_1954) | [GH13_16](http://www.cazy.org/GH13_16.html) | [GH13_16](http://www.cazy.org/GH13_16.html) | [GH13](http://www.cazy.org/GH13.html) |  | H7F36_RS09855 | Trehalose synthase |
|  | [NZ_CP060296.1_1956](https://bcb.unl.edu/dbCAN2/domain.php?jobid=2021101664237&gene=NZ_CP060296.1_1956) | [GH13_10](http://www.cazy.org/GH13_10.html) | CBM48+GH13  _10 | GH13+CBM48 | COG0296 |  | Malto-oligosyltrehalose  trehalohydrolase |
|  | [NZ_CP060296.1_1957](https://bcb.unl.edu/dbCAN2/domain.php?jobid=2021101664237&gene=NZ_CP060296.1_1957) | GH77+GH13_26 | GH13_26+GH77 | GH13+GH77 |  | H7F36_RS09870 | Malto-oligosyltrehalose synthase |
| ***V.* sp. 38R** | [NZ_CP062121.1_675](https://bcb.unl.edu/dbCAN2/domain.php?jobid=2021101605147&gene=NZ_CP062121.1_675) | [GH15](http://www.cazy.org/GH15.html) | [GH15](http://www.cazy.org/GH15.html) | [GH15](http://www.cazy.org/GH15.html) | COG3387 |  | α, α-trehalase |
|  | [NZ_CP062121.1_676](https://bcb.unl.edu/dbCAN2/domain.php?jobid=2021101605147&gene=NZ_CP062121.1_676) | [GT20](http://www.cazy.org/GT20.html) | [GT20](http://www.cazy.org/GT20.html) | [GT20](http://www.cazy.org/GT20.html) | COG0380/  COG1877 |  | α, α-trehalose-phosphate synthase/  trehalose-6-P phosphatase |
|  | [NZ_CP062121.1_2401](https://bcb.unl.edu/dbCAN2/domain.php?jobid=2021101605147&gene=NZ_CP062121.1_2401) | [GH13_16](http://www.cazy.org/GH13_16.html) | [GH13_16](http://www.cazy.org/GH13_16.html) | [GH13](http://www.cazy.org/GH13.html) |  | IG196_RS12165 | Trehalose synthase |
|  | [NZ_CP062121.1_2404](https://bcb.unl.edu/dbCAN2/domain.php?jobid=2021101605147&gene=NZ_CP062121.1_2404) | [GH13_10](http://www.cazy.org/GH13_10.html) | CBM48+GH13  _10 | GH13+CBM48 | COG0296 |  | Malto-oligosyltrehalose  trehalohydrolase |
|  | [NZ_CP062121.1_2405](https://bcb.unl.edu/dbCAN2/domain.php?jobid=2021101605147&gene=NZ_CP062121.1_2405) | GH77+GH13_26 | GH13_26+GH77 | GH13+GH77 |  | IG196_RS12165 | Malto-oligosyltrehalose synthase |
| ***V.* sp. PDNC026** | [NZ_CP014517.1_1280](https://bcb.unl.edu/dbCAN2/domain.php?jobid=2021101614941&gene=NZ_CP014517.1_1280) | [GT20](http://www.cazy.org/GT20.html) | [GT20](http://www.cazy.org/GT20.html) | [GT20](http://www.cazy.org/GT20.html) | COG0380/  COG1877 |  | α, α-trehalose-phosphate synthase/  trehalose-6-P phosphatase |
|  | [NZ_CP014517.1_2051](https://bcb.unl.edu/dbCAN2/domain.php?jobid=2021101614941&gene=NZ_CP014517.1_2051) | [GH37](http://www.cazy.org/GH37.html) | [GH37](http://www.cazy.org/GH37.html) | [GH37](http://www.cazy.org/GH37.html) |  |  | α, α-trehalase |
|  | [NZ_CP014517.1_3266](https://bcb.unl.edu/dbCAN2/domain.php?jobid=2021101614941&gene=NZ_CP014517.1_3266) | GH77+GH13_26 | GH13_26+GH77 | GH13+GH77 |  | JVX96_RS22395 | Malto-oligosyltrehalose synthase |
|  | [NZ_CP014517.1_3267](https://bcb.unl.edu/dbCAN2/domain.php?jobid=2021101614941&gene=NZ_CP014517.1_3267) | [GH13_10](http://www.cazy.org/GH13_10.html) | CBM48+GH13_10 | GH13+CBM48 | COG0296 |  | Malto-oligosyltrehalose  trehalohydrolase |
|  | [NZ_CP014517.1_3269](https://bcb.unl.edu/dbCAN2/domain.php?jobid=2021101614941&gene=NZ_CP014517.1_3269) | [GH13_16](http://www.cazy.org/GH13_16.html) | [GH13_16](http://www.cazy.org/GH13_16.html) | [GH13](http://www.cazy.org/GH13.html) |  | JVX96_RS22415 | Trehalose synthase |
|  | [NZ_CP014517.1_784](https://bcb.unl.edu/dbCAN2/domain.php?jobid=2021101614941&gene=NZ_CP014517.1_784) | [GH15](http://www.cazy.org/GH15.html) | [GH15](http://www.cazy.org/GH15.html) | [GH15](http://www.cazy.org/GH15.html) | COG3387 |  | α, α-trehalase |
| ***V.* sp. RA8** | [NZ_LR594662.1_1150](https://bcb.unl.edu/dbCAN2/domain.php?jobid=2021101620555&gene=NZ_LR594662.1_1150) | [GT20](http://www.cazy.org/GT20.html) | [GT20](http://www.cazy.org/GT20.html) | [GT20](http://www.cazy.org/GT20.html) | COG0380/  COG1877 |  | α, α-trehalose-phosphate synthase/  trehalose-6-P phosphatase |
|  | [NZ_LR594662.1_1151](https://bcb.unl.edu/dbCAN2/domain.php?jobid=2021101620555&gene=NZ_LR594662.1_1151) | [GH15](http://www.cazy.org/GH15.html) | [GH15](http://www.cazy.org/GH15.html) | [GH15](http://www.cazy.org/GH15.html) | COG3387 |  | α, α-trehalase |
|  | [NZ_LR594662.1_1309](https://bcb.unl.edu/dbCAN2/domain.php?jobid=2021101620555&gene=NZ_LR594662.1_1309) | [GH37](http://www.cazy.org/GH37.html) | [GH37](http://www.cazy.org/GH37.html) | [GH37](http://www.cazy.org/GH37.html) | COG1626 |  | α, α-trehalase |
|  | [NZ_LR594662.1_1408](https://bcb.unl.edu/dbCAN2/domain.php?jobid=2021101620555&gene=NZ_LR594662.1_1408) | GH77GH13_26 | GH13_26+GH77 | GH13+GH77 |  | E5P3_RS07070 | Malto-oligosyltrehalose synthase |
|  | [NZ_LR594662.1_1409](https://bcb.unl.edu/dbCAN2/domain.php?jobid=2021101620555&gene=NZ_LR594662.1_1409) | [GH13_10](http://www.cazy.org/GH13_10.html) | CBM48+GH13_10 | GH13+CBM48 | COG0296 |  | Malto-oligosyltrehalose  trehalohydrolase |
|  | [NZ_LR594662.1_1412](https://bcb.unl.edu/dbCAN2/domain.php?jobid=2021101620555&gene=NZ_LR594662.1_1412) | [GH13_16](http://www.cazy.org/GH13_16.html) | [GH13_16](http://www.cazy.org/GH13_16.html) | [GH13](http://www.cazy.org/GH13.html) |  | E5P3_RS07090 | Trehalose synthase |
| ***V.* sp. RKNM96** | [NZ_CP046508.1_1044](https://bcb.unl.edu/dbCAN2/domain.php?jobid=2021101623054&gene=NZ_CP046508.1_1044) | [GH15](http://www.cazy.org/GH15.html) | [GH15](http://www.cazy.org/GH15.html) | [GH15](http://www.cazy.org/GH15.html) | COG3387 |  | α, α-trehalase |
|  | [NZ_CP046508.1_1043](https://bcb.unl.edu/dbCAN2/domain.php?jobid=2021101623054&gene=NZ_CP046508.1_1043) | [GT20](http://www.cazy.org/GT20.html) | [GT20](http://www.cazy.org/GT20.html) | [GT20](http://www.cazy.org/GT20.html) | COG0380/  COG1877 |  | α, α-trehalose-phosphate synthase/  trehalose-6-P phosphatase |
|  | [NZ_CP046508.1_3300](https://bcb.unl.edu/dbCAN2/domain.php?jobid=2021101623054&gene=NZ_CP046508.1_3300) | GH77+GH13_26 | GH13_26+GH77 | GH13+GH77 |  | GNX71_RS16745 | Malto-oligosyltrehalose synthase |
|  | [NZ_CP046508.1_3301](https://bcb.unl.edu/dbCAN2/domain.php?jobid=2021101623054&gene=NZ_CP046508.1_3301) | [GH13_10](http://www.cazy.org/GH13_10.html) | [GH13_10](http://www.cazy.org/GH13_10.html) | GH13+CBM48 |  | GNX71_RS16750 | Malto-oligosyltrehalose  trehalohydrolase |
|  | [NZ_CP046508.1_3304](https://bcb.unl.edu/dbCAN2/domain.php?jobid=2021101623054&gene=NZ_CP046508.1_3304) | [GH13_16](http://www.cazy.org/GH13_16.html) | [GH13_16](http://www.cazy.org/GH13_16.html) | [GH13](http://www.cazy.org/GH13.html) |  | GNX71_RS16765 | Trehalose synthase |
|  | [NZ_CP046508.1_5867](https://bcb.unl.edu/dbCAN2/domain.php?jobid=2021101623054&gene=NZ_CP046508.1_5867) | [GH37](http://www.cazy.org/GH37.html) | [GH37](http://www.cazy.org/GH37.html) | [GH37](http://www.cazy.org/GH37.html) | COG1626 |  | α, α-trehalase |
| ***V.* sp. SRS16** | [NZ_LR594666.1_151](https://bcb.unl.edu/dbCAN2/domain.php?jobid=2021101640719&gene=NZ_LR594666.1_151) | [GH37](http://www.cazy.org/GH37.html) | [GH37](http://www.cazy.org/GH37.html) | [GH37](http://www.cazy.org/GH37.html) |  | E5P2_RS00755 | α, α-trehalase |
|  | [NZ_LR594666.1_3013](https://bcb.unl.edu/dbCAN2/domain.php?jobid=2021101640719&gene=NZ_LR594666.1_3013) | [GH13_16](http://www.cazy.org/GH13_16.html) | [GH13_16](http://www.cazy.org/GH13_16.html) | [GH13](http://www.cazy.org/GH13.html) |  | E5P2_RS15270 | Trehalose synthase |
|  | [NZ_LR594666.1_3016](https://bcb.unl.edu/dbCAN2/domain.php?jobid=2021101640719&gene=NZ_LR594666.1_3016) | [GH13_10](http://www.cazy.org/GH13_10.html) | CBM48+GH13_10 | GH13+CBM48 | COG0296 |  | Malto-oligosyltrehalose  trehalohydrolase |
|  | [NZ_LR594666.1_3017](https://bcb.unl.edu/dbCAN2/domain.php?jobid=2021101640719&gene=NZ_LR594666.1_3017) | GH77+GH13_26 | GH13_26+GH77 | GH13+GH77 |  | E5P2_RS15290 | Malto-oligosyltrehalose synthase |
|  | [NZ_LR594666.1_967](https://bcb.unl.edu/dbCAN2/domain.php?jobid=2021101640719&gene=NZ_LR594666.1_967) | [GT20](http://www.cazy.org/GT20.html) | [GT20](http://www.cazy.org/GT20.html) | [GT20](http://www.cazy.org/GT20.html) | COG0380/  COG1877 |  | α, α-trehalose-phosphate synthase/  trehalose-6-P phosphatase |
| ***V.* sp. WDL1** | [NZ_LR594689.1_4320](https://bcb.unl.edu/dbCAN2/domain.php?jobid=2021101642654&gene=NZ_LR594689.1_4320) | [GT20](http://www.cazy.org/GT20.html) | [GT20](http://www.cazy.org/GT20.html) | [GT20](http://www.cazy.org/GT20.html) | COG0380/  COG1877 |  | α, α-trehalose-phosphate synthase/  trehalose-6-P phosphatase |
|  | [NZ_LR594689.1_4321](https://bcb.unl.edu/dbCAN2/domain.php?jobid=2021101642654&gene=NZ_LR594689.1_4321) | [GH15](http://www.cazy.org/GH15.html) | [GH15](http://www.cazy.org/GH15.html) | [GH15](http://www.cazy.org/GH15.html) | COG3387 |  | α, α-trehalase |
|  | [NZ_LR594689.1_4477](https://bcb.unl.edu/dbCAN2/domain.php?jobid=2021101642654&gene=NZ_LR594689.1_4477) | GH77+GH13_26 | GH13_26+GH77 | GH13+GH77 |  | E5P1_RS22675 | Malto-oligosyltrehalose synthase |
|  | [NZ_LR594689.1_4478](https://bcb.unl.edu/dbCAN2/domain.php?jobid=2021101642654&gene=NZ_LR594689.1_4478) | [GH13_10](http://www.cazy.org/GH13_10.html) | CBM48+GH13_10 | GH13+CBM48 | COG00296 |  | Malto-oligosyltrehalose  trehalohydrolase |
|  | [NZ_LR594689.1_4481](https://bcb.unl.edu/dbCAN2/domain.php?jobid=2021101642654&gene=NZ_LR594689.1_4481) | [GH13_16](http://www.cazy.org/GH13_16.html) | [GH13_16](http://www.cazy.org/GH13_16.html) | [GH13](http://www.cazy.org/GH13.html) |  | E5P1_RS22695 | Trehalose synthase |
| ***V.* sp. PBL-E5** | [NZ_LR594671.1_947](https://bcb.unl.edu/dbCAN2/domain.php?jobid=2021101665023&gene=NZ_LR594671.1_947) | [GT20](http://www.cazy.org/GT20.html) | [GT20](http://www.cazy.org/GT20.html) | [GT20](http://www.cazy.org/GT20.html) | COG0380/  COG1877 |  | α, α-trehalose-phosphate synthase/  trehalose-6-P phosphatase |
|  | [NZ_LR594671.1_146](https://bcb.unl.edu/dbCAN2/domain.php?jobid=2021101665023&gene=NZ_LR594671.1_146) | [GH37](http://www.cazy.org/GH37.html) | [GH37](http://www.cazy.org/GH37.html) | [GH37](http://www.cazy.org/GH37.html) | COG1626 |  | α, α-trehalase |
|  | [NZ_LR594671.1_3009](https://bcb.unl.edu/dbCAN2/domain.php?jobid=2021101665023&gene=NZ_LR594671.1_3009) | [GH13_16](http://www.cazy.org/GH13_16.html) | [GH13_16](http://www.cazy.org/GH13_16.html) | [GH13](http://www.cazy.org/GH13.html) |  | WDLP6_RS15260 | Trehalose synthase |
|  | [NZ_LR594671.1_3012](https://bcb.unl.edu/dbCAN2/domain.php?jobid=2021101665023&gene=NZ_LR594671.1_3012) | [GH13_10](http://www.cazy.org/GH13_10.html) | CBM48+GH13_10 | GH13+CBM48 | COG0296 |  | Malto-oligosyltrehalose  trehalohydrolase |
|  | [NZ_LR594671.1_3013](https://bcb.unl.edu/dbCAN2/domain.php?jobid=2021101665023&gene=NZ_LR594671.1_3013) | GH77+GH13_26 | GH13_26+GH77 | GH13+GH77 |  | WDLP6_RS15280 | Malto-oligosyltrehalose synthase |
| ***V.* sp. PBL-H6** | [NZ_LR594659.1_1011](https://bcb.unl.edu/dbCAN2/domain.php?jobid=2021101672045&gene=NZ_LR594659.1_1011) | [GT20](http://www.cazy.org/GT20.html) | [GT20](http://www.cazy.org/GT20.html) | [GT20](http://www.cazy.org/GT20.html) | COG0380/  COG1877 |  | α, α-trehalose-phosphate synthase/  trehalose-6-P phosphatase |
|  | [NZ_LR594659.1_1012](https://bcb.unl.edu/dbCAN2/domain.php?jobid=2021101672045&gene=NZ_LR594659.1_1012) | [GH15](http://www.cazy.org/GH15.html) | [GH15](http://www.cazy.org/GH15.html) | [GH15](http://www.cazy.org/GH15.html) | COG3387 |  | α, α-trehalase |
|  | [NZ_LR594659.1_1181](https://bcb.unl.edu/dbCAN2/domain.php?jobid=2021101672045&gene=NZ_LR594659.1_1181) | GH77+GH13_26 | GH13_26+GH77 | GH13+GH77 |  | G3W89_RS05945 | Malto-oligosyltrehalose synthase |
|  | [NZ_LR594659.1_1182](https://bcb.unl.edu/dbCAN2/domain.php?jobid=2021101672045&gene=NZ_LR594659.1_1182) | [GH13_10](http://www.cazy.org/GH13_10.html) | CBM48+GH13_10 | GH13+CBM48 | COG00296 |  | Malto-oligosyltrehalose  trehalohydrolase |
|  | [NZ_LR594659.1_1185](https://bcb.unl.edu/dbCAN2/domain.php?jobid=2021101672045&gene=NZ_LR594659.1_1185) | [GH13_16](http://www.cazy.org/GH13_16.html) | [GH13_16](http://www.cazy.org/GH13_16.html) | [GH13](http://www.cazy.org/GH13.html) |  | G3W89_RS05965 | Trehalose synthase |
|  | [NZ_LR594659.1_499](https://bcb.unl.edu/dbCAN2/domain.php?jobid=2021101672045&gene=NZ_LR594659.1_499) | [GH37](http://www.cazy.org/GH37.html) | [GH37](http://www.cazy.org/GH37.html) | [GH37](http://www.cazy.org/GH37.html) | COG1626 |  | α, α-trehalase |
| ***V.* sp. PMC12** | [NZ_CP027773.1_2804](https://bcb.unl.edu/dbCAN2/domain.php?jobid=20211107205220&gene=NZ_CP027773.1_2804) | [GH15](http://www.cazy.org/GH15.html) | [GH15](http://www.cazy.org/GH15.html) | [GH15](http://www.cazy.org/GH15.html) | COG3387 |  | α, α-trehalase |
|  | [NZ_CP027773.1_2805](https://bcb.unl.edu/dbCAN2/domain.php?jobid=20211107205220&gene=NZ_CP027773.1_2805) | [GT20](http://www.cazy.org/GT20.html) | [GT20](http://www.cazy.org/GT20.html) | [GT20](http://www.cazy.org/GT20.html) | COG1877 |  | α, α-trehalose-phosphate synthase/  trehalose-6-P phosphatase |
|  | [NZ_CP027773.1_421](https://bcb.unl.edu/dbCAN2/domain.php?jobid=20211107205220&gene=NZ_CP027773.1_421) | [GH13_16](http://www.cazy.org/GH13_16.html) | [GH13_16](http://www.cazy.org/GH13_16.html) | [GH13](http://www.cazy.org/GH13.html) |  | C4F17_RS02150 | Trehalose synthase |
|  | [NZ_CP027773.1_424](https://bcb.unl.edu/dbCAN2/domain.php?jobid=20211107205220&gene=NZ_CP027773.1_424) | [GH13_10](http://www.cazy.org/GH13_10.html) | CBM48+GH13_10 | GH13+CBM48 | COG0296 |  | Malto-oligosyltrehalose  trehalohydrolase |
|  | [NZ_CP027773.1_425](https://bcb.unl.edu/dbCAN2/domain.php?jobid=20211107205220&gene=NZ_CP027773.1_425) | GH77+GH13_26 | GH13_26+GH77 | GH13+GH77 |  | C4F17_RS02170 | Malto-oligosyltrehalose synthase |
| ***V.* sp. PBS-H4** | [NZ_LR594675.1_1023](https://bcb.unl.edu/dbCAN2/domain.php?jobid=2021101674825&gene=NZ_LR594675.1_1023) | [GT20](http://www.cazy.org/GT20.html) | [GT20](http://www.cazy.org/GT20.html) | [GT20](http://www.cazy.org/GT20.html) | COG0380/  COG1877 |  | α, α-trehalose-phosphate synthase/  trehalose-6-P phosphatase |
|  | [NZ_LR594675.1_1024](https://bcb.unl.edu/dbCAN2/domain.php?jobid=2021101674825&gene=NZ_LR594675.1_1024) | [GH15](http://www.cazy.org/GH15.html) | [GH15](http://www.cazy.org/GH15.html) | [GH15](http://www.cazy.org/GH15.html) | COG3387 |  | α, α-trehalase |
|  | [NZ_LR594675.1_1218](https://bcb.unl.edu/dbCAN2/domain.php?jobid=2021101674825&gene=NZ_LR594675.1_1218) | GH77+GH13_26 | GH13_26+GH77 | GH13+GH77 |  | E5CHR_RS06130 | Malto-oligosyltrehalose synthase |
|  | [NZ_LR594675.1_1219](https://bcb.unl.edu/dbCAN2/domain.php?jobid=2021101674825&gene=NZ_LR594675.1_1219) | [GH13_10](http://www.cazy.org/GH13_10.html) | CBM48+GH13_10 | GH13+CBM48 | COG0296 |  | Malto-oligosyltrehalose  trehalohydrolase |
|  | [NZ_LR594675.1_1222](https://bcb.unl.edu/dbCAN2/domain.php?jobid=2021101674825&gene=NZ_LR594675.1_1222) | [GH13_16](http://www.cazy.org/GH13_16.html) | [GH13_16](http://www.cazy.org/GH13_16.html) | [GH13](http://www.cazy.org/GH13.html) |  | E5CHR_RS06150 | Trehalose synthase |
|  | [NZ_LR594675.1_474](https://bcb.unl.edu/dbCAN2/domain.php?jobid=2021101674825&gene=NZ_LR594675.1_474) | [GH37](http://www.cazy.org/GH37.html) | [GH37](http://www.cazy.org/GH37.html) | [GH37](http://www.cazy.org/GH37.html) | COG1626 |  | α, α -trehalase |
| ***V. paradoxus* CSUSB** | [NZ_CP046622.1_1986](https://bcb.unl.edu/dbCAN2/domain.php?jobid=2021101711322&gene=NZ_CP046622.1_1986) | [GH13_16](http://www.cazy.org/GH13_16.html) | [GH13_16](http://www.cazy.org/GH13_16.html) | [GH13](http://www.cazy.org/GH13.html) |  | GOQ09_RS10075 | Trehalose synthase |
|  | [NZ_CP046622.1_2000](https://bcb.unl.edu/dbCAN2/domain.php?jobid=2021101711322&gene=NZ_CP046622.1_2000) | [GH13_10](http://www.cazy.org/GH13_10.html) | CBM48+GH13_10 | GH13+CBM48 |  | GOQ09_RS10090 | Malto-oligosyltrehalose  trehalohydrolase |
|  | [NZ_CP046622.1_2001](https://bcb.unl.edu/dbCAN2/domain.php?jobid=2021101711322&gene=NZ_CP046622.1_2001) | GH77+GH13_26 | GH13_26+GH77 | GH13+GH77 |  | GOQ09_RS10095 | Malto-oligosyltrehalose synthase |
|  | [NZ_CP046622.1_927](https://bcb.unl.edu/dbCAN2/domain.php?jobid=2021101711322&gene=NZ_CP046622.1_927) | [GT20](http://www.cazy.org/GT20.html) | [GT20](http://www.cazy.org/GT20.html) | [GT20](http://www.cazy.org/GT20.html) | COG0380/  COG1877 |  | α, α-trehalose-phosphate synthase/  trehalose-6-P phosphatase |
|  | [NZ_CP046622.1_928](https://bcb.unl.edu/dbCAN2/domain.php?jobid=2021101711322&gene=NZ_CP046622.1_928) | [GH15](http://www.cazy.org/GH15.html) | [GH15](http://www.cazy.org/GH15.html) | [GH15](http://www.cazy.org/GH15.html) | COG3387 |  | α, α-trehalase |
| ***V. paradoxus*  5C-2** | [NZ_CP045644.1_126](https://bcb.unl.edu/dbCAN2/domain.php?jobid=2021101652827&gene=NZ_CP045644.1_126) | [GH13_16](http://www.cazy.org/GH13_16.html) | [GH13_16](http://www.cazy.org/GH13_16.html) | [GH13](http://www.cazy.org/GH13.html) |  | GFK26_RS26950 | Trehalose synthase |
|  | [NZ_CP045644.1_351](https://bcb.unl.edu/dbCAN2/domain.php?jobid=2021101652827&gene=NZ_CP045644.1_351) | [GT20](http://www.cazy.org/GT20.html) | [GT20](http://www.cazy.org/GT20.html) | [GT20](http://www.cazy.org/GT20.html) | COG0380/  COG1877 |  | α, α-trehalose-phosphate synthase/ trehalose-6-P-phosphatase |
|  | [NZ_CP045644.1_5308](https://bcb.unl.edu/dbCAN2/domain.php?jobid=2021101652827&gene=NZ_CP045644.1_5308) | GH77+GH13_26 | GH13_26+GH77 | GH13+GH77 |  | GFK26_RS26930 | Malto-oligosyltrehalose synthase |
|  | [NZ_CP045644.1_5309](https://bcb.unl.edu/dbCAN2/domain.php?jobid=2021101652827&gene=NZ_CP045644.1_5309) | [GH13_10](http://www.cazy.org/GH13_10.html) | CBM48+GH13_10 | GH13+CBM48 | COG0296 |  | Malto-oligosyltrehalose  trehalohydrolase |
|  | [NZ_CP045644.1_763](https://bcb.unl.edu/dbCAN2/domain.php?jobid=2021101652827&gene=NZ_CP045644.1_763) | [GH15](http://www.cazy.org/GH15.html) | [GH15](http://www.cazy.org/GH15.html) | [GH15](http://www.cazy.org/GH15.html) | COG3387 |  | α, α-trehalase |
| ***V. paradoxus*  VAI-C** | [CP063166.1_1182](https://bcb.unl.edu/dbCAN2/domain.php?jobid=2021101711334&gene=CP063166.1_1182) | [GT20](http://www.cazy.org/GT20.html) | [GT20](http://www.cazy.org/GT20.html) | [GT20](http://www.cazy.org/GT20.html) | COG0380/  COG1877 |  | α, α-trehalose-phosphate synthase/trehalose-6-P phosphatase |
|  | [CP063166.1_1183](https://bcb.unl.edu/dbCAN2/domain.php?jobid=2021101711334&gene=CP063166.1_1183) | [GH15](http://www.cazy.org/GH15.html) | [GH15](http://www.cazy.org/GH15.html) | [GH15](http://www.cazy.org/GH15.html) | COG3387 |  | α, α-trehalase |
|  | [CP063166.1_847](https://bcb.unl.edu/dbCAN2/domain.php?jobid=2021101711334&gene=CP063166.1_847) | GH77+GH13_26 | GH13_26+GH77 | GH13+GH77 |  | INQ48_04250 | Malto-oligosyltrehalose synthase |
|  | [CP063166.1_848](https://bcb.unl.edu/dbCAN2/domain.php?jobid=2021101711334&gene=CP063166.1_848) | [GH13_10](http://www.cazy.org/GH13_10.html) | CBM48+GH13_10 | GH13+CBM48 | COG0296 |  | Malto-oligosyltrehalose  trehalohydrolase |
|  | [CP063166.1_851](https://bcb.unl.edu/dbCAN2/domain.php?jobid=2021101711334&gene=CP063166.1_851) | [GH13_16](http://www.cazy.org/GH13_16.html) | [GH13_16](http://www.cazy.org/GH13_16.html) | [GH13](http://www.cazy.org/GH13.html) |  | INQ48_04270 | Trehalose synthase |
| ***V. paradoxus*  EPS** | [NC_014931.1_1049](https://bcb.unl.edu/dbCAN2/domain.php?jobid=20211107205531&gene=NC_014931.1_1049) | [GT20](http://www.cazy.org/GT20.html) | [GT20](http://www.cazy.org/GT20.html) | [GT20](http://www.cazy.org/GT20.html) | COG0380/  COG1877 |  | α, α-trehalose-phosphate synthase/trehalose-6-P phosphatase |
|  | [NC_014931.1_2999](https://bcb.unl.edu/dbCAN2/domain.php?jobid=20211107205531&gene=NC_014931.1_2999) | [GH13_16](http://www.cazy.org/GH13_16.html) | [GH13_16](http://www.cazy.org/GH13_16.html) | [GH13](http://www.cazy.org/GH13.html) |  | VARPA_RS14990 | Trehalose synthase |
|  | [NC_014931.1_3002](https://bcb.unl.edu/dbCAN2/domain.php?jobid=20211107205531&gene=NC_014931.1_3002) | [GH13_10](http://www.cazy.org/GH13_10.html) | CBM48+GH13_10 | GH13+CBM48 | COG0296 |  | Malto-oligosyltrehalose  trehalohydrolase |
|  | [NC_014931.1_3003](https://bcb.unl.edu/dbCAN2/domain.php?jobid=20211107205531&gene=NC_014931.1_3003) | GH77+GH13_26 | GH13_26+GH77 | GH13+GH77 |  | VARPA_RS15010 | Malto-oligosyltrehalose synthase |
|  | [NC_014931.1_1050](https://bcb.unl.edu/dbCAN2/domain.php?jobid=20211107205531&gene=NC_014931.1_1050) | [GH15](http://www.cazy.org/GH15.html) | [GH15](http://www.cazy.org/GH15.html) | [GH15](http://www.cazy.org/GH15.html) | COG3387 |  | α, α-trehalase |
| ***V. paradoxus*  B4** | [NC_022247.1_1026](https://bcb.unl.edu/dbCAN2/domain.php?jobid=20211107210259&gene=NC_022247.1_1026) | [GT20](http://www.cazy.org/GT20.html) | [GT20](http://www.cazy.org/GT20.html) | [GT20](http://www.cazy.org/GT20.html) | COG0380/  COG1877 |  | α, α-trehalose-phosphate synthase/trehalose-6-P phosphatase |
|  | [NC_022247.1_1027](https://bcb.unl.edu/dbCAN2/domain.php?jobid=20211107210259&gene=NC_022247.1_1027) | [GH15](http://www.cazy.org/GH15.html) | [GH15](http://www.cazy.org/GH15.html) | [GH15](http://www.cazy.org/GH15.html) | COG3387 |  | α, α-trehalase |
|  | [NC_022247.1_2085](https://bcb.unl.edu/dbCAN2/domain.php?jobid=20211107210259&gene=NC_022247.1_2085) | [GH13_16](http://www.cazy.org/GH13_16.html) | [GH13_16](http://www.cazy.org/GH13_16.html) | [GH13](http://www.cazy.org/GH13.html) |  | VAPA_RS10395 | Trehalose synthase |
|  | [NC_022247.1_2088](https://bcb.unl.edu/dbCAN2/domain.php?jobid=20211107210259&gene=NC_022247.1_2088) | [GH13_10](http://www.cazy.org/GH13_10.html) | CBM48+GH13_10 | GH13+CBM48 | COG0296 |  | Malto-oligosyltrehalose  trehalohydrolase |
|  | [NC_022247.1_2089](https://bcb.unl.edu/dbCAN2/domain.php?jobid=20211107210259&gene=NC_022247.1_2089) | GH77+GH13_26 | GH13_26+GH77 | GH13+GH77 |  | VAPA_RS10415 | Malto-oligosyltrehalose synthase |
| ***V. boronicumulans* J1** | [NZ_CP023284.1_1012](https://bcb.unl.edu/dbCAN2/domain.php?jobid=20211107210559&gene=NZ_CP023284.1_1012) | [GH15](http://www.cazy.org/GH15.html) | [GH15](http://www.cazy.org/GH15.html) | [GH15](http://www.cazy.org/GH15.html) | COG3387 |  | α, α-trehalase |
|  | [NZ_CP023284.1_1013](https://bcb.unl.edu/dbCAN2/domain.php?jobid=20211107210559&gene=NZ_CP023284.1_1013) | [GT20](http://www.cazy.org/GT20.html) | [GT20](http://www.cazy.org/GT20.html) | [GT20](http://www.cazy.org/GT20.html) | COG0380/  COG1877 |  | α, α-trehalose-phosphate synthase/trehalose-6-P phosphatase |

“*” represents our strains studied in this paper.

**Supplementary Table S6**. Pairwise comparisons of query strains vs. type strain genomes.

| **Query strain** | **Subject strain** | **dDDH (d4, in %)** | **C.I. (d4, in %)** |
| --- | --- | --- | --- |
| '*Variovorax* sp. PAMC28562' (ASM1430373v1) | *‘Variovorax* sp. PAMC 28711 (ASM157726v1) | 23.3 | [21.0 - 25.8] |
|  | *Variovorax boronicumulans* NBRC 103145 | 22.9 | [20.6 - 25.3] |
|  | *Variovorax paradoxus* NBRC 15149 | 22.8 | [20.5 - 25.2] |
|  | *Variovorax beijingensis* 502T | 22.7 | [20.4 - 25.1] |
|  | *Variovorax gossypii* DSM 100435 | 22.6 | [20.3 - 25.0] |
|  | *Variovorax guangxiensis* DSM 27352 | 22.5 | [20.2 - 25.0] |
|  | *Variovorax soli* NBRC 106424 | 20.9 | [18.6 - 23.3] |
|  | *Xylophilus ampelinus* CECT 7646 | 20.5 | [18.2 - 22.9] |
|  | *Xenophilus azovorans* DSM 13620 | 20.4 | [18.2 - 22.8] |
|  | *Pseudacidovorax intermedius* DSM 21352 | 20.2 | [18.0 - 22.6] |
|  | *Pseudorhodoferax soli* DSM 21634 | 19.8 | [17.6 - 22.2] |
|  | *Pseudorhodoferax aquiterrae* KCTC23314 | 19.6 | [17.4 - 22.0] |
|  | *Acidovorax anthurii* DSM 16745 | 19.6 | [17.4 - 22.0] |
|  | *Rhodoferax saidenbachensis* ED16 | 19.2 | [17.0 - 21.5] |
|  | *Rhodoferax aquaticus* GR-4 | 19.2 | [17.0 - 21.6] |
|  | *Caenimonas koreensis* DSM 17982 | 18.9 | [16.7 - 21.3] |
|  | *Pelomonas puraquae* CCUG 52769 | 18.6 | [16.4 - 21.0] |
|  | *Limnohabitans parvus* CIP109845 | 18.2 | [16.1 - 20.6] |
| '*Variovorax* sp. PAMC26660' (ASM1430299v1) | *Variovorax boronicumulans* NBRC 103145 | 32.1 | [29.7 - 34.7] |
|  | *Variovorax gossypii* DSM 100435 | 31.6 | [29.2 - 34.1] |
|  | *Variovorax guangxiensis* DSM 27352 | 31.5 | [29.1 - 34.0] |
|  | *Variovorax paradoxus* NBRC 15149 | 31.4 | [29.0 - 33.9] |
|  | *Variovorax beijingensis* 502T | 31.2 | [28.8 - 33.7] |
|  | *‘Variovorax* sp. PAMC 28711' (ASM157726v1) | 24 | [21.7 - 26.5] |
|  | *Variovorax soli* NBRC 106424 | 22.8 | [20.5 - 25.3] |
|  | *‘Variovorax* sp. PAMC28562' (ASM1430373v1) | 22.7 | [20.4 - 25.2] |
|  | *Xenophilus azovorans* DSM 13620 | 22.1 | [19.9 - 24.6] |
|  | *Pseudacidovorax intermedius* DSM 21352 | 21.8 | [19.5 - 24.2] |
|  | *Xylophilus ampelinus* CECT 7646 | 21.6 | [19.3 - 24.0] |
|  | *Acidovorax anthurii* DSM 16745 | 21.2 | [19.0 - 23.6] |
|  | *Pseudorhodoferax aquiterrae* KCTC23314 | 21.1 | [18.8 - 23.5] |
|  | *Pseudorhodoferax soli* DSM 21634 | 21 | [18.7 - 23.4] |
|  | *Caenimonas koreensis* DSM 17982 | 19.9 | [17.7 - 22.3] |
|  | *Limnohabitans parvus* CIP109845 | 19.5 | [17.3 - 21.9] |
|  | *Rhodoferax saidenbachensis* ED16 | 19.4 | [17.2 - 21.8] |
|  | *Rhodoferax aquaticus* GR-4 | 19.3 | [17.1 - 21.7] |
|  | *Pelomonas puraquae* CCUG 52769 | 19.2 | [17.0 - 21.6] |
| '*Variovorax* sp. PAMC 28711' (ASM157726v1) | *Variovorax boronicumulans* NBRC 103145 | 24.5 | [22.2 - 27.0] |
|  | *Variovorax paradoxus* NBRC 15149 | 24.5 | [22.2 - 26.9] |
|  | *Variovorax beijingensis* 502T | 24.3 | [22.0 - 26.8] |
|  | *Variovorax gossypii* DSM 100435 | 24.1 | [21.8 - 26.6] |
|  | *Variovorax* g*uangxiensis* DSM 27352 | 24 | [21.7 - 26.4] |
|  | *Variovorax soli* NBRC 106424 | 21.9 | [19.6 - 24.3] |
|  | *Xenophilus azovorans* DSM 13620 | 21.6 | [19.4 - 24.1] |
|  | *Xylophilus ampelinus* CECT 7646 | 21.2 | [18.9 - 23.6] |
|  | *Pseudacidovorax intermedius* DSM 21352 | 21.1 | [18.8 - 23.5] |
|  | *Pseudorhodoferax aquiterrae KCTC23314* | 20.6 | [18.3 - 23.0] |
|  | *Pseudorhodoferax soli* DSM 21634 | 20.5 | [18.3 - 23.0] |
|  | *Acidovorax anthurii* DSM 16745 | 20.5 | [18.3 - 22.9] |
|  | *Caenimonas koreensis* DSM 17982 | 19.3 | [17.1 - 21.7] |
|  | *Rhodoferax saidenbachensis* ED16 | 19.2 | [17.0 - 21.6] |
|  | *Limnohabitans parvus* CIP109845 | 19.1 | [16.9 - 21.5] |
|  | *Pelomonas puraquae* CCUG 52769 | 18.9 | [16.7 - 21.3] |
|  | *Rhodoferax aquaticus* GR-4 | 18.8 | [16.6 - 21.2] |

**Supplementary Fig. 1.** ML (Maximum Likelihood) tree of the *Variovorax* dataset inferred under the GTR+GAMMA model. The tree is rooted at the midpoint and branches are scaled in terms of the expected number of substitutions per site. The numbers above the branches are support values when larger than 60% from ML (left) and MP (right) bootstrapping. The tree was inferred through TYGS via the DSMZ gene phylogeny pipeline available under [https://ggdc.dsmz.de/phylogeny-service.php](https://ggdc.dsmz.de/phylogeny-service#.php).


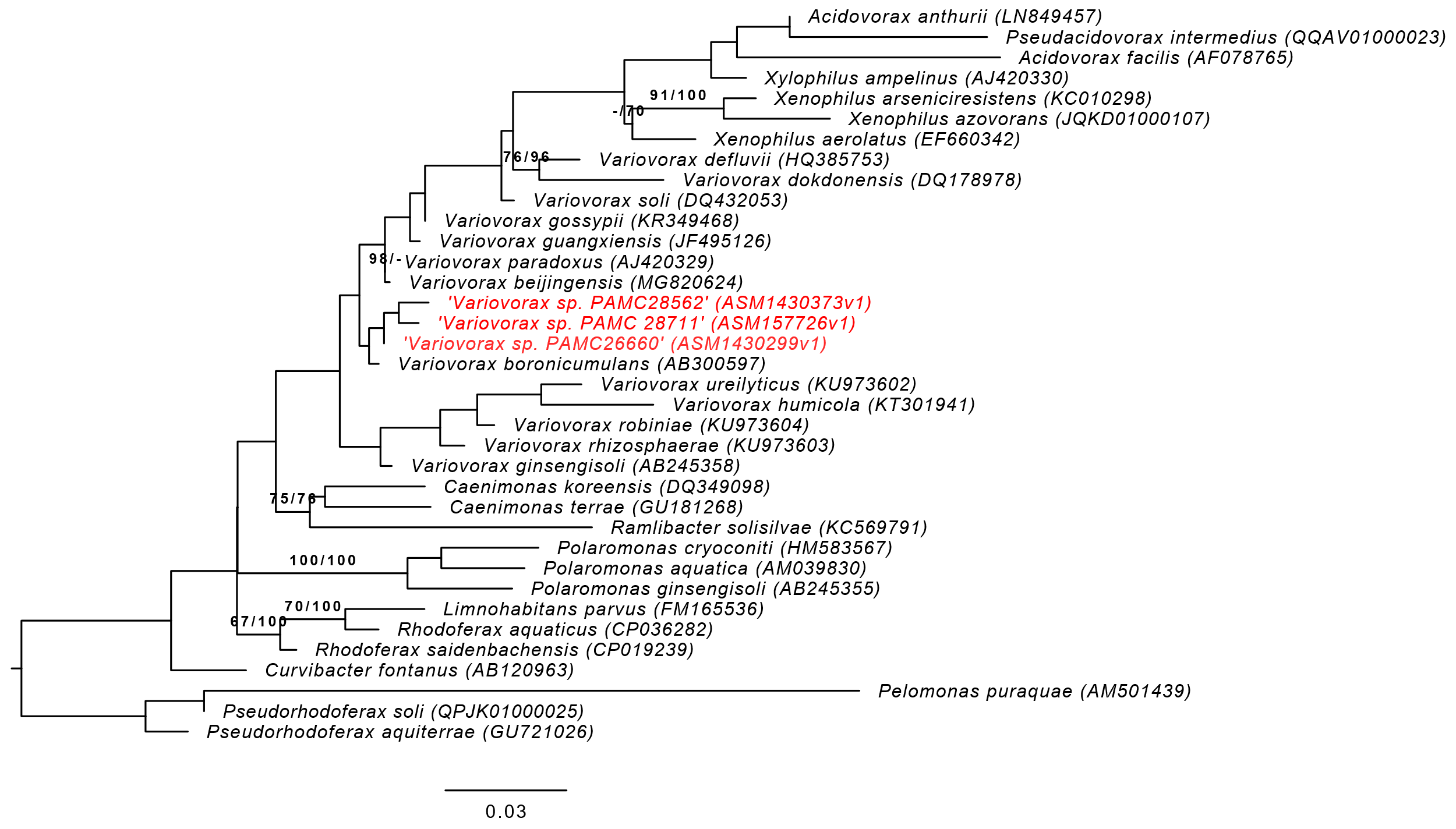

Supplement: Supplementary Materials — Supplementary Table S1: genomic information of the three complete metagenomic assembled genomes of Variovorax sp. Supplementary Table S2: comparison of glycoside hydrolases (GHs) families and subfamilies among the complete genome of Variovorax strains. Supplementary Table S3: comparison of glycosyl transferases (GTs) families and subfamilies among the complete genome of Variovorax strains. Supplementary Table S4: comparison of carbohydrate esterases (CEs), carbohydrate-binding modules (CBMs), auxiliary activities (AAs), and polysaccharide lyases (PLs) families and subfamilies among the complete genome of Variovorax strains. Supplementary Table S5: the CAZyme subfamilies of trehalose metabolism of the complete Variovorax strains by dbCAN2 meta server, Prokka annotation, and GenBank locus tag. Supplementary Table S6: pairwise comparisons of query strains vs. type strain genomes. Supplementary Figure 1: ML (Maximum Likelihood) tree of the Variovorax dataset inferred under the GTR + GAMMA model. The tree is rooted at the midpoint, and branches are scaled in terms of the expected number of substitutions per site. The numbers above the branches are support values when larger than 60% from ML (left) and MP (right) bootstrapping. The tree was inferred through TYGS via the DSMZ gene phylogeny pipeline available under https://ggdc.dsmz.de/phylogeny-service.php. [file 5067074.f1.docx]
